# Research progress on targeting autophagy pathways with medicinal plants and their active metabolites for the treatment of heart failure

**DOI:** 10.3389/fphar.2026.1908479

**Published:** 2026-09-04

**Authors:** Yan Dong, Yanzhen Wang, Xiaoqing Kou

**Affiliations:** 1 Cardiothoracic Surgery, Lanzhou First People’s Hospital, Lanzhou, Gansu, China; 2 Cardiovascular Disease Research Institute, Lanzhou First People’s Hospital, Lanzhou, Gansu, China; 3 The First Clinical Medical College, Lanzhou University, Lanzhou, Gansu, China

**Keywords:** active metabolites, autophagy pathway, Chinese herbal compound preparations, heart failure, medicinal plants, single-botanical drug extracts

## Abstract

Despite the therapeutic promise of natural plants in preclinical models, their clinical value for heart failure (HF) remains unconfirmed and requires rigorous validation through well-designed trials. HF severely impairs quality of life, and natural plant therapies—known for their multi-metabolites and multi-target effects—have been explored as potential treatments *via* modulation of autophagy, inhibition of inflammation, and reduction of oxidative stress. However, several critical issues remain unresolved: the broad-spectrum activity raises concerns about target specificity and off-target effects. Current evidence largely relies on correlative observations rather than direct mechanistic validation. The assumption that natural products are inherently safer than synthetic drugs is flawed due to dose-dependent toxicity and the absence of long-term clinical data. And the reductionist isolation of active metabolites may overlook the synergistic actions of whole herbal preparations. Moreover, while modulation of multiple autophagy-related pathways is theoretically superior, existing data stem primarily from basic or observational studies rather than from rigorously designed randomized controlled trials. Chinese herbal formulas exhibit “multi-metabolite, multi-target, multi-pathway” actions that cannot be explained by a single pathway, and the transition from traditional use to evidence-based therapy faces challenges in standardization, quality control, and dose optimization. This review critically examines the ethnopharmacological use of natural plants in HF through autophagy regulation, drawing on traditional medicine classics and databases including PubMed, China National Knowledge Infrastructure (CNKI), Web of Science, and Wanfang. Our key findings indicate that medicinal plants offer advantages in multi-target regulation and safety, but these potential benefits must be interpreted with caution. Claims of “significant clinical efficacy” should not be overgeneralized. Thus, rigorous methodologies and large-scale clinical trials are urgently needed to confirm whether natural plant therapies can truly translate into tangible benefits for HF patients.

## Graphical Abstract

**Figure:**
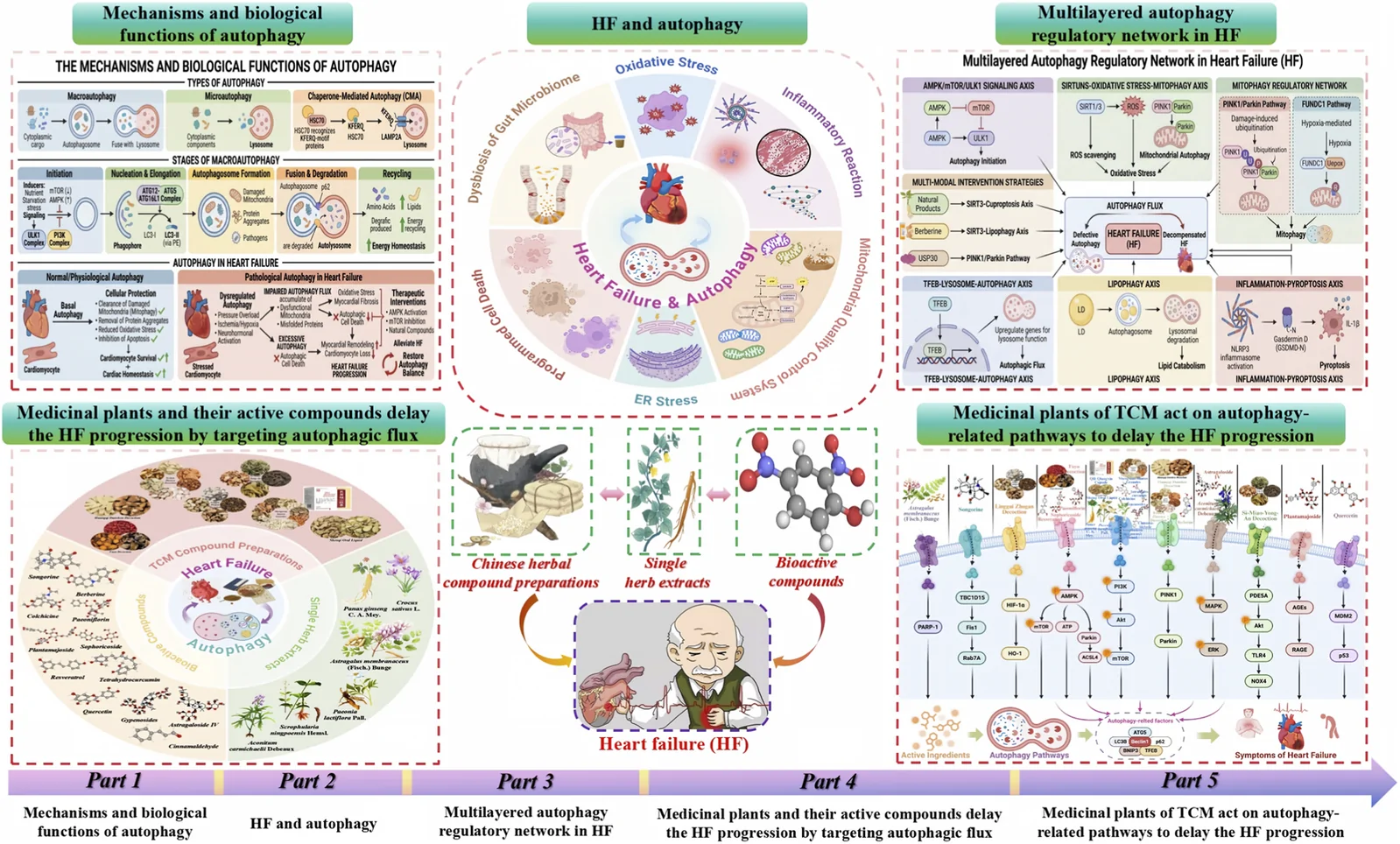

## 1 Introduction

Heart failure (HF) represents the terminal manifestation of multiple cardiovascular diseases and predominantly affects middle-aged and older adults. Its typical features include reduced exercise tolerance, dyspnea, fluid retention, such as peripheral edema and pulmonary congestion, and fatigue; systemic manifestations such as renal dysfunction and cognitive impairment may also occur (Fragin and Stephens, 2024). With the acceleration of global population aging, HF has become a major public health challenge in cardiovascular medicine, with a continuously rising prevalence and disease burden. Epidemiological data show that the incidence of HF increases markedly after 65 years of age and is slightly higher in men than in women. Currently, more than 64 million people worldwide live with HF. In China, the number of cases is increasing particularly rapidly because of the large population base and accelerated aging, and the continually growing patient population underscores the severity of HF as a major health problem (Boer et al., 2026). In terms of treatment, angiotensin receptor-neprilysin inhibitors (ARNIs) and beta-blockers have been shown to improve outcomes in patients with heart failure with reduced ejection fraction (HFrEF) and have become foundational therapies. Current pharmacological strategies mainly aim to suppress excessive neuroendocrine activation and reduce fluid retention, often through combined use of diuretics, sodium-glucose cotransporter 2 (SGLT2) inhibitors, and related agents. For advanced patients who respond poorly to drug therapy or have cardiac dyssynchrony or malignant arrhythmias, device-based and surgical interventions, including cardiac resynchronization therapy (CRT), implantable cardioverter-defibrillators (ICDs), and heart transplantation, are important adjunctive options and can significantly improve quality of life (Guo et al., 2025). Nevertheless, existing treatments mainly focus on alleviating symptoms and slowing disease progression, they still cannot reverse damaged myocardial structures or completely halt disease deterioration. These limitations highlight the need for therapies that can modify the disease course and for more precise, individualized treatment strategies.

Traditional Chinese medicine (TCM) employs complex formulas composed of multiple bioactive metabolites that act on multiple targets. In recent years, its use as an adjunctive therapy for HF has attracted increasing attention, and related basic and clinical evidence has continued to accumulate (Wu et al., 2023). However, the broad-spectrum activity of these multi-component formulas raises legitimate concerns regarding target specificity, particularly when modulating autophagy—a highly regulated and context-dependent cellular process. Autophagy plays dual roles in cardiomyocytes: moderate activation can clear damaged organelles and protect against stress, whereas excessive or insufficient autophagy may exacerbate cardiac dysfunction. Non-selective modulation of autophagy by TCM metabolites could inadvertently affect off-target pathways or disrupt the fine balance of autophagic flux, potentially leading to unforeseen adverse effects. Therefore, while multi-target intervention offers theoretical advantages for complex diseases like HF, it also necessitates rigorous mechanistic dissection to identify the precise molecular targets and to distinguish beneficial effects from nonspecific actions. According to TCM theory, HF is often classified under categories such as “heart water (a condition of fluid retention affecting cardiac function),” “heart impediment (obstruction or stasis of heart vessels),” and “edema (accumulation or swelling of generalized fluid)”. Its pathogenesis commonly involves deficiency of heart qi and yang, blood stasis, and water retention, and treatment should be based on syndrome differentiation. Classical formulas such as Zhenwu Decoction and Baoyuan Decoction combined with Tingli Dazao Xiefei Decoction have been widely used in clinical practice and have been preliminarily validated in standardized studies (Lan et al., 2024). Mechanistic studies indicate that certain Chinese herbal metabolites can improve cardiac function by regulating autophagy pathways. For example, salvianolic acid B, derived from *Salvia miltiorrhiza* Bge., can promote cardiomyocyte mitophagy by activating the PINK1/Parkin signaling pathway, thereby attenuating myocardial injury (Hu et al., 2020). Baicalin can induce autophagy by regulating the AMPK/mTOR signaling pathway and suppress cardiomyocyte apoptosis and fibrosis (Cheng et al., 2024). Clinical data show that Chinese herbal compound preparations, such as Qili Qiangxin capsules, can significantly improve symptom scores, cardiac ejection fraction, and exercise tolerance in patients with HF and reduce rehospitalization due to HF worsening (Sun et al., 2016). In addition, when compound preparations are combined with conventional anti-HF drugs, such as ARNIs and beta-blockers, they may further alleviate clinical symptoms, improve quality of life, and potentially reduce the required doses of diuretics or vasoactive agents in some patients (Jung et al., 2024; Wu J. et al., 2025). Together, these findings suggest that TCM offers distinctive advantages in HF treatment, including multi-target and multi-pathway regulation of disease mechanisms, synergistic therapeutic effects, and relatively fewer adverse reactions, thereby providing more diverse and individualized treatment strategies for patients with HF. Nevertheless, the aforementioned concerns regarding target specificity underscore the need for continued investigation into the precise molecular mechanisms and context-dependent actions of TCM constituents to fully realize their therapeutic potential while minimizing risks.

From a pathological perspective, the core features of HF include progressive cardiomyocyte loss, myocardial fibrotic remodeling, and systolic/diastolic dysfunction. Mitochondrial injury and abnormal accumulation of toxic misfolded proteins are key factors driving HF deterioration (Hinton et al., 2024). Therefore, elucidating how cells remove damaged organelles and toxic protein aggregates is crucial for clarifying the pathogenesis of HF and identifying novel therapeutic targets. Autophagy is a highly conserved intracellular degradation and recycling system that forms autophagosomes and delivers cargo to lysosomes for degradation. It includes macroautophagy, microautophagy, and chaperone-mediated autophagy (CMA) (Liu S. et al., 2023). Extensive evidence indicates that autophagy dysfunction is closely associated with HF progression. Impaired autophagic flux leads to the accumulation of dysfunctional mitochondria, aggravates oxidative stress, and promotes cardiomyocyte apoptosis and fibrosis, further weakening the heart’s pumping capacity. In addition, key signaling molecules that regulate autophagy, including AMPK, mTOR, and Beclin1, are abnormally expressed during HF progression, while mutations in cardiomyopathy-related genes, such as MYBPC3 and TTN, may also interfere with normal function of the autophagy-lysosome pathway, suggesting a causal role for autophagy impairment in HF development (Du et al., 2020). Therefore, in-depth investigation of autophagy regulation in HF not only helps reveal its pathological nature but also suggests that targeting autophagy pathways may represent a promising therapeutic strategy.

Recent studies indicate that autophagy is closely related to the onset and progression of HF. In addition, metabolic disturbances caused by HF are tightly associated with the mechanisms regulating autophagy (Ott, 2024; Tang Y. et al., 2024). On this basis, this review systematically describes the regulatory mechanisms and related biological processes of autophagy, discusses key signaling molecules involved in the pathological process of HF, and systematically summarizes medicinal plants that can improve HF symptoms by modulating autophagy, highlighting the potential of these natural products as innovative therapeutic strategies.

## 2 Literature search and methods

**FIGURE 1:**
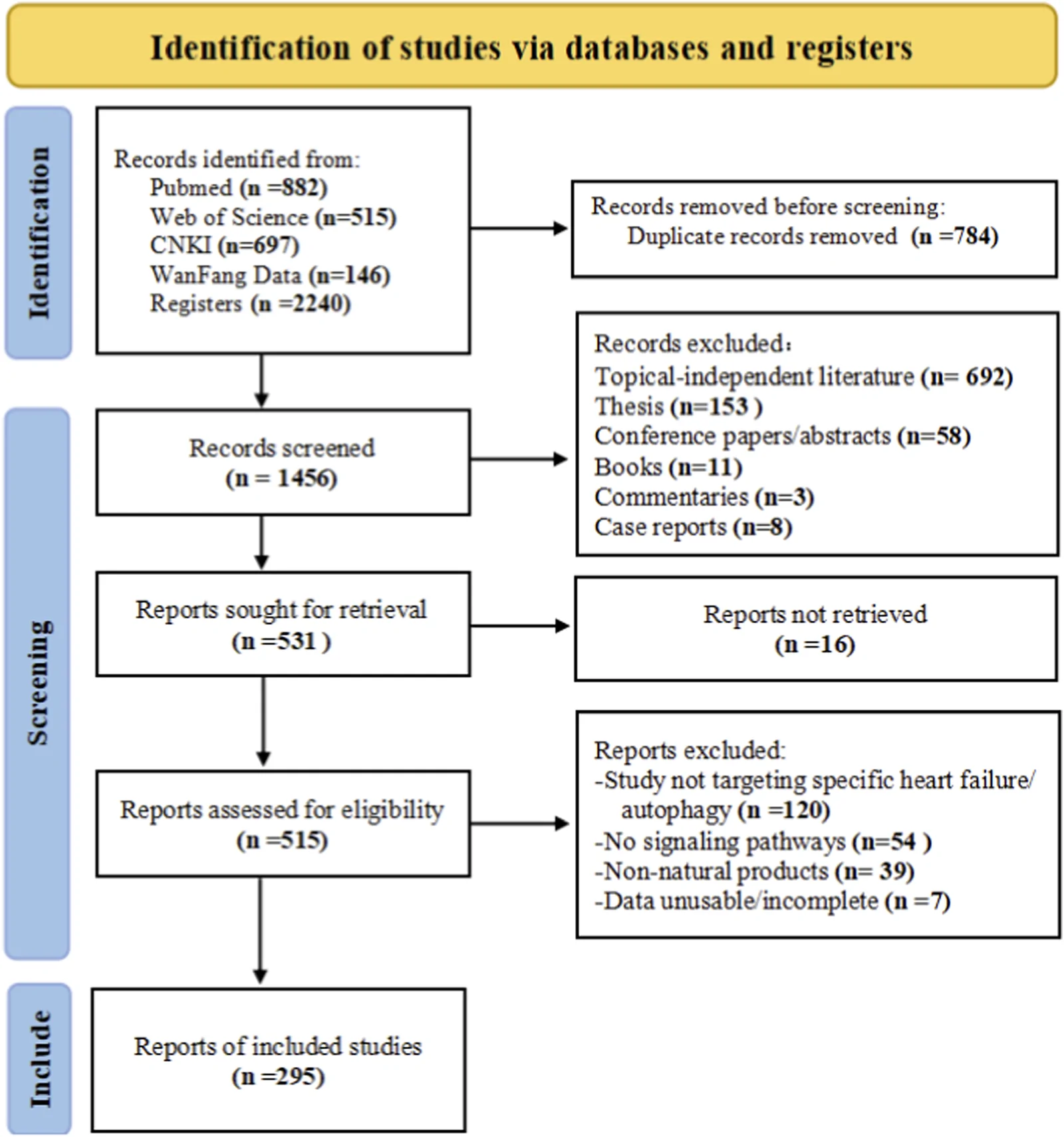
The literature retrieval and screening process based on the PRISMA principles.

This study systematically reviewed *in vitro*, *in vivo*, and clinical evidence regarding the protective effects of medicinal plants against HF and their molecular mechanisms, with a focus on autophagy-related signaling pathways. PubMed, China National Knowledge Infrastructure (CNKI), Web of Science, and Wanfang Data were searched from database inception to June 2026. Search terms included HF, autophagy (including “autophagy,” “macroautophagy,” “mitophagy,” or “lipophagy”), and natural products (including “medicinal plants,” “TCM,” “plant extracts,” or “bioactive metabolites”). Reference lists of relevant articles were also manually screened. Inclusion and exclusion criteria were explicitly defined: original studies (both preclinical and clinical) reporting autophagy-related mechanistic data—including *in vitro*, *in vivo*, or clinical investigations—and high-quality reviews were included. Preclinical studies encompassed cell-based assays and animal models, while clinical studies comprised randomized controlled trials, prospective or retrospective cohort studies. Exclusion criteria comprised irrelevant literature, dissertations, conference abstracts, books, case reports, commentaries, and studies not published in English or Chinese. After deduplication and preliminary screening, 2,240 records were identified. Two independent reviewers screened titles, abstracts, and full texts. The disagreements were resolved through discussion or consultation with a third reviewer. Ultimately, 295 studies met the inclusion criteria (Figure 1). Quality assessment was performed to evaluate the risk of bias in the included studies. For animal experiments, the SYRCLE’s Risk of Bias tool was applied. For randomized controlled trials, the Cochrane Risk of Bias (RoB 2.0) tool was used. And for non-randomized clinical studies, the ROBINS-I tool was employed. The quality of each study was independently assessed by two reviewers, with discrepancies resolved by consensus. Publication bias was assessed visually using funnel plots for outcomes with sufficient data (≥10 studies), supplemented by Egger’s test to detect asymmetry where applicable. Although this review was not registered in PROSPERO, we adhered to the Preferred Reporting Items for Systematic Reviews and Meta-Analyses (PRISMA) guidelines to ensure transparency and completeness of reporting. Extracted data included the names of medicinal plants and metabolites, experimental models, key findings, and autophagy-related mechanisms. Given the heterogeneity of interventions and outcome measures, a narrative synthesis approach was adopted. All plant species were taxonomically verified through the MPNS portal (http://mpns.kew.org/mpns-portal/), and complete scientific names are provided.

## 3 Biological functions of autophagy in HF

“Autophagy” (from the Greek, meaning “self-eating”) is a highly conserved lysosomal degradation pathway in eukaryotic cells. It removes dysfunctional proteins, damaged organelles, and pathogens, maintains energy homeostasis through nutrient recycling, and ensures cellular quality control. This process is precisely regulated by autophagy-related genes (ATGs) and key signaling pathways such as mTOR and AMPK (Ohsumi, 2014). Studies have shown that autophagy has a typical “double-edged sword” property: basal autophagy protects cells, whereas dysregulated autophagy can induce or exacerbate multiple diseases (Yang and Klionsky, 2020). The canonical macroautophagy process mainly involves several key steps: (1) initiation and nucleation mediated by the ULK1 and PI3K complexes. (2) Phagophore elongation and autophagosome formation promoted by ubiquitin-like conjugation systems, such as the ATG12-ATG5-ATG16L1 complex and the LC3-PE system. (3) Fusion of autophagosomes with lysosomes, enabling degradation and recycling of the cargo (Kirat et al., 2023). According to the mechanism by which substrates are delivered to lysosomes, autophagy can be divided into three types. Macroautophagy, which mediates bulk degradation through autophagosomes. Microautophagy, in which the lysosomal membrane directly invaginates to engulf substrates. And CMA, in which proteins containing KFERQ-like motifs are specifically recognized and transported through the LAMP2A receptor (Yamamoto and Matsui, 2024).

**FIGURE 2:**
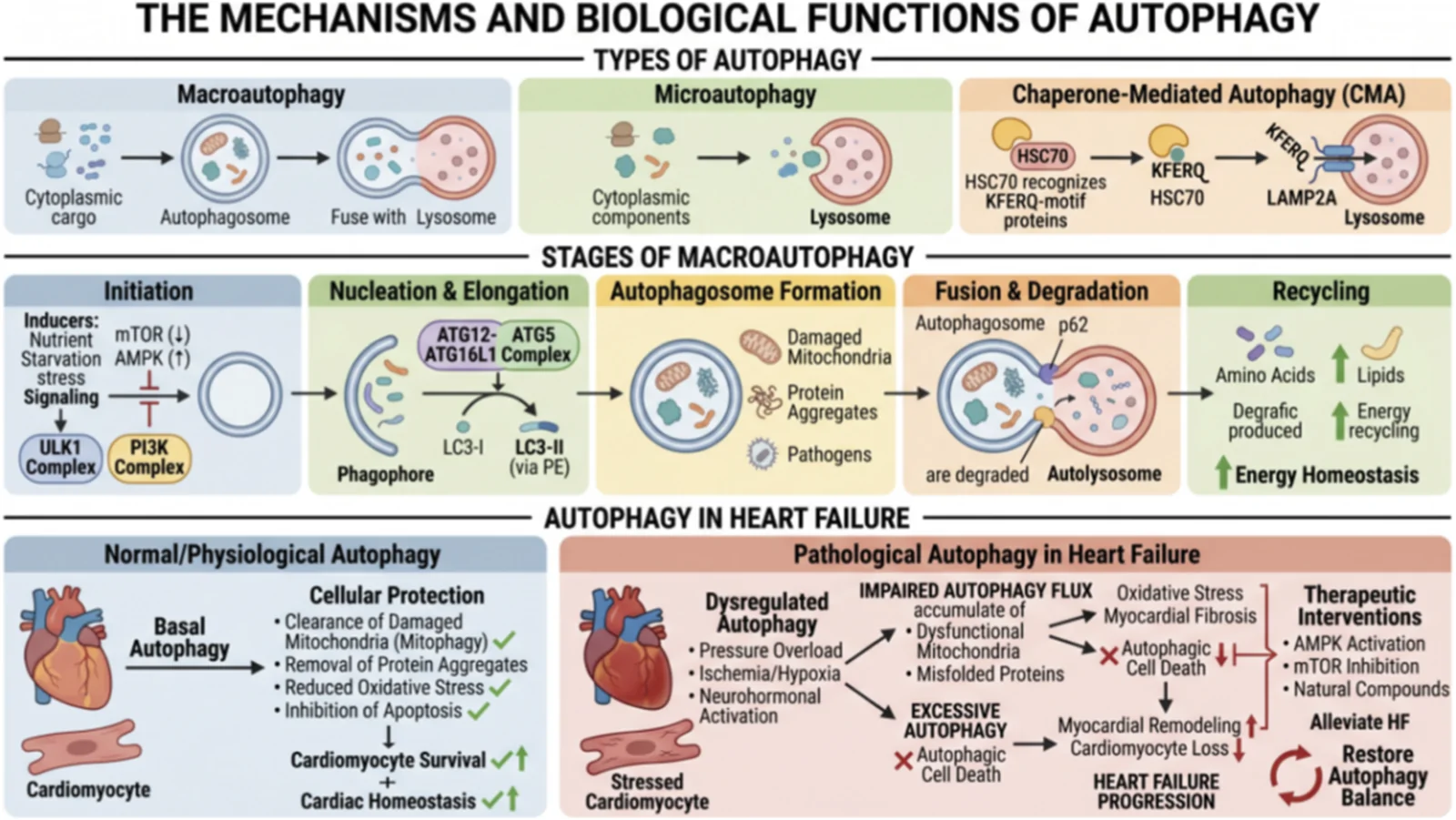
The mechanisms and biological functions of autophagy contributing to HF. The diagram summarizes the types of autophagy, the stages of macroautophagy, and the autophagy in HF.

Research on autophagy in disease has become a frontier topic in biomedicine. Dysfunctional autophagy is closely associated with various neurodegenerative diseases, such as Parkinson’s disease and Alzheimer’s disease, and plays complex roles in cancer, infection, and metabolic disorders. In the pathological progression of HF, autophagy also exerts critical and bidirectional regulatory effects. Because cardiomyocytes are terminally differentiated cells, their autophagic function is essential for maintaining cardiac homeostasis. Under physiological conditions, basal autophagy helps remove damaged mitochondria and toxic protein aggregates and suppresses oxidative stress and apoptosis. However, during HF progression, sustained pressure overload, ischemia/hypoxia, and excessive neuroendocrine activation can lead to impaired autophagic flux or excessive autophagy activation. On the one hand, impaired autophagy causes the accumulation of dysfunctional mitochondria, namely mitophagy impairment, and misfolded proteins, thereby accelerating cardiomyocyte loss and myocardial fibrotic remodeling. On the other hand, excessive autophagy may induce self-digestion of cardiomyocytes and further worsen cardiac function (Du et al., 2020). Preclinical studies suggest that moderate regulation of autophagy, for example through AMPK activation, mTOR inhibition, or intervention with specific natural products, can restore autophagic flux, reduce cardiomyocyte injury, and delay HF progression (Li X. et al., 2019; Tang et al., 2026; Yang R.-Z. et al., 2025). Therefore, in-depth clarification of the precise regulatory mechanisms of autophagy in HF not only helps reveal the pathological essence of the disease but also provides new directions for developing autophagy-targeted therapeutic strategies. Taken together, autophagy is a fundamental cellular process that maintains functional homeostasis and overall health. In the complex syndrome of HF, balanced regulation of autophagy is particularly important and has considerable potential for clinical translation (Figure 2).

## 4 HF and autophagy

### 4.1 Role of autophagy in the pathological process of HF

Autophagy also plays a key role in the pathological progression of HF, and its defects may occur at multiple stages of autophagic flux. Imbalance in upstream signaling pathways, such as mTOR and AMPK, can disrupt normal initiation of autophagy. Altered expression or function of ATG proteins may impair autophagosome formation. At later stages, including autophagosome-lysosome fusion and substrate degradation, are often blocked by reduced activity of the lysosomal hydrolase, impaired integrity of the lysosomal membrane, or defects in vesicular transport. Notably, misfolded proteins under pathological conditions, such as aggregated ubiquitinated proteins, and damaged organelles can serve as autophagic substrates and can also conversely inhibit the autophagic process. Studies have shown that persistent accumulation of toxic protein aggregates in cardiomyocytes may interfere with the expression of the lysosomal membrane protein LAMP2, suppress the autophagy-lysosome pathway, and form a positive-feedback loop that further aggravates proteotoxic stress and cardiomyocyte loss (Corsetti et al., 2019).

Mitochondrial dysfunction is a typical pathological feature of HF. Loss of function or downregulation of HF-related genes, such as Parkin, PINK1, and FUNDC1, leads to abnormal accumulation of damaged mitochondria and excessive ROS production, ultimately causing disordered energy metabolism and cardiomyocyte apoptosis (Yang, 2025). Exogenous injurious factors, such as doxorubicin, ischemia/reperfusion injury, and pressure overload, can further aggravate mitochondrial damage by inducing mitochondrial complex I inhibition, calcium overload, or oxidative stress, and can disrupt autophagic flux in the failing heart (Ravindran and Gustafsson, 2025). Meanwhile, genetic factors further highlight the central role of autophagy in HF. Studies have found that mutations in sarcomeric protein genes, such as MYBPC3 and MYH7, can cause abnormal mechanical stress in cardiomyocytes and indirectly interfere with autophagosome formation and trafficking (de Frutos et al., 2022; Tudurachi et al., 2023). Mutations in lysosome-related genes, such as LAMP2-FLOT2, directly cause lysosomal dysfunction and blocked autophagic degradation, which is particularly prominent during septic cardiomyopathy (Shao et al., 2025). In addition, abnormal expression of trafficking-related proteins, such as VPS35, may disrupt retrograde vesicular transport and affect the normal dynamic convergence of autophagosomes and lysosomes (Shi et al., 2026).

Collectively, these findings confirm the “double-edged sword” nature of autophagy in HF: restoration of efficient and complete autophagic flux helps remove toxic proteins and damaged mitochondria and exerts cardioprotective effects, whereas excessive, mistimed, or incomplete autophagy activation, especially when lysosomal clearance is impaired, may aggravate metabolic stress, promote pyroptosis or apoptosis, and accelerate deterioration of cardiac function. Therefore, strategies such as enhancing autophagic flux, restoring lysosomal function, or specifically activating mitophagy, including the PINK1/Parkin and FUNDC1 pathways, through small-molecule metabolites, gene therapy, or natural products are being actively explored as potential disease-modifying therapeutic approaches. Future work should further clarify the dynamic evolution of different forms of autophagy, such as macroautophagy, mitophagy, and lipophagy, and their individual stages during the onset and progression of HF, in order to achieve precise regulation tailored to the pathological context.

### 4.2 Potential pathological links between autophagy and HF

**FIGURE 3:**
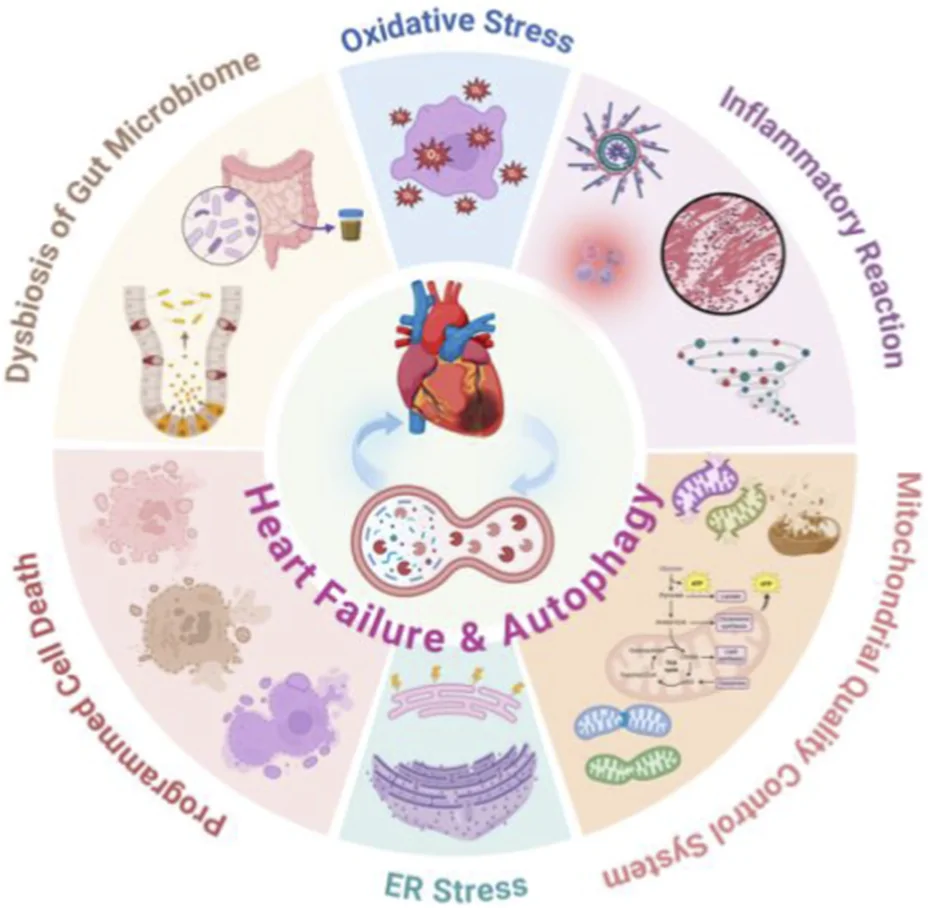
The potential pathological link between HF and autophagy, including inflammatory response, mitochondrial quality control system, oxidative stress, ER stress, dysbiosis of gut microbiome, necrotic apoptosis, pyroptosis, and ferroptosis.

HF arises from complex molecular disturbances and dysregulated cell death pathways. Autophagy participates in multiple HF-related pathological processes, including inflammatory responses, impaired mitochondrial quality control, dysbiosis of gut microbiome, oxidative stress, endoplasmic reticulum (ER) stress, necroptosis, pyroptosis, and ferroptosis (Figure 3). A more detailed analysis of these interactions will deepen mechanistic understanding and facilitate the development of translational therapies that target autophagy within broader immunometabolic networks.

#### 4.2.1 Autophagy and sterile inflammation

Autophagy and sterile inflammation interact closely and bidirectionally, jointly influencing HF progression. Autophagy alleviates excessive inflammatory responses and cardiomyocyte pyroptosis by clearing damaged mitochondria, toxic protein aggregates, ROS, and other DAMPs, and by modulating innate immune signaling, including inhibition of NLRP3 inflammasome activation and maturation and release of downstream cytokines such as IL-1β and IL-18. Conversely, inflammatory signals can also regulate autophagy activity. For example, the proinflammatory cytokines TNF-α and IL-6 may inhibit autophagic flux through NF-κB or JAK/STAT signaling pathways, whereas certain anti-inflammatory or reparative signals can enhance autophagy to maintain intracellular homeostasis in cardiomyocytes (Gupta et al., 2025). In HF, impaired autophagy leads to abnormal accumulation of damaged mitochondria, oxidized mitochondrial DNA (mtDNA), and misfolded proteins. These DAMPs activate inflammatory signaling in cardiac-resident macrophages, fibroblasts, and cardiomyocytes through innate immune pathways, including Toll-like receptors (TLRs) and the NLRP3 inflammasome, thereby aggravating sterile myocardial inflammation, fibrotic remodeling, and deterioration of cardiac function. Conversely, a chronic inflammatory environment further impairs autophagic flux, for example by inhibiting nuclear translocation of the transcription factor TFEB and lysosomal function, forming a pathological cycle of positive-feedback (Papamichail et al., 2023). In addition, HF-related genetic or pathological factors may affect both sides of this regulatory loop. For example, pressure overload or doxorubicin-induced myocardial injury can impair autophagosome-lysosome fusion while enhancing NF-κB signaling and proinflammatory responses (Tang J. et al., 2024). Mutations in lysosome-associated genes (such as LAMP2) directly impair the autophagy-lysosomal degradation process and exacerbate inflammatory activation of macrophages (Tapia et al., 2025).

However, the assertion that “autophagy activation inherently exhibits anti-inflammatory effects” requires careful evaluation, as this relationship is highly context-dependent. Autophagy may produce markedly different effects in different cell types, such as cardiomyocytes, fibroblasts, and macrophages. Moderate autophagy enhancement in cardiomyocytes can remove toxic metabolites, whereas excessive autophagy in macrophages may affect antigen presentation and alter inflammatory phenotypes. In addition, many studies still rely on static markers such as LC3-II, p62, and LAMP1/2, which cannot distinguish enhanced autophagy initiation from impaired autophagic flux. Moreover, acute ischemia/reperfusion or endotoxin injection models may exaggerate signaling relationships that only partially reflect the chronic and heterogeneous process of sterile inflammation observed in human HF. More reliable evidence requires cell-type-specific measurements of autophagic flux, such as tandem fluorescent-tagged LC3, and longitudinal study designs rather than reliance on marker changes at a single time point.

#### 4.2.2 Autophagy and disruption of mitochondrial quality-control system

Mitochondrial quality control depends on mitophagy, mitochondrial biogenesis, and balanced fusion-fission dynamics, among which macroautophagy is the main mechanism for clearing dysfunctional mitochondria (Liu B.-H. et al., 2024). In HF, this regulatory network is often disrupted. The PINK1/Parkin signaling pathway plays a central role in mitophagy, and its loss of function or downregulation leads to damaged mitochondrial accumulation, increased ROS levels, bioenergetic crisis, activation of apoptotic pathways, and progressive cardiomyocyte loss (Qiu et al., 2019). In addition, mitochondrial dysfunction can in turn impair autophagy. For example, ATP depletion and oxidative stress hinder autophagosome formation and autophagosome-lysosome fusion (Yoshii et al., 2024). Conversely, impaired autophagy aggravates mitochondrial homeostatic imbalance. Broad inhibition of autophagy results in abnormal mitochondrial accumulation, whereas mutations in genes related to mitophagy and trafficking, such as FBXO7 and VPS35, directly impair mitochondrial renewal (Zhong et al., 2023; Manders et al., 2025). Notably, pathological protein aggregates, such as misfolded ubiquitinated proteins and DAMPs released by senescent mitochondria, can damage mitochondria and inhibit PINK1/Parkin-mediated mitophagy, thereby forming a self-reinforcing pathological cycle (Fung et al., 2025). Furthermore, pathological stimuli such as doxorubicin, ischemia/reperfusion injury, or pressure overload can induce mitochondrial complex I inhibition and lysosomal dysfunction while disrupting autophagy-lysosome function, thereby reproducing key features of HF (Chen et al., 2026; Jiang H. et al., 2025).

However, the optimistic therapeutic outlook surrounding enhanced mitophagy warrants rigorous scrutiny. This optimism is largely based on acute ischemia/reperfusion or drug-induced models, such as doxorubicin models, which mainly reflect acute mitochondrial damage and autophagic stress and may not accurately reproduce the chronic, progressive, and multifactorial pathological process observed in human HF. In addition, commonly used assessment indicators, including Parkin recruitment, the LC3-II/I ratio, or p62 levels, may be misleading when lysosomal clearance remains the rate-limiting step, both increased autophagy initiation and blocked autophagic flux can manifest as elevated LC3-II. Finally, accelerating mitochondrial removal without ensuring compensatory mitochondrial biogenesis may harm cardiomyocytes, which have extremely high energy demands. In these cells, the boundary between neuroprotection, or cardioprotection, and bioenergetic failure is very blurred. Therefore, future studies must combine cell-type-specific dynamic monitoring of autophagic flux, multi-time-point evaluation, and assessment of synchronized regulation of mitochondrial biogenesis to more accurately determine the true value of mitophagy as a therapeutic target in HF.

#### 4.2.3 Autophagy and oxidative stress

Oxidative stress arises from excessive ROS production and imbalance in antioxidant systems and can cause oxidative damage to cardiomyocyte lipids, proteins, and DNA (Sies, 2015). Autophagy and oxidative stress are closely and bidirectionally regulated, they interact in the pathogenesis of HF and jointly promote progressive cardiomyocyte loss and deterioration of cardiac function. On the one hand, the failing heart often exhibits marked oxidative stress because of mitochondrial respiratory chain dysfunction, excessive neuroendocrine activation, and inflammatory responses. Excessive production of ROS, such as hydroxyl radicals (·OH) and hydrogen peroxide (H_2_O_2_), can oxidize and modify autophagy-related proteins, such as ATG4 and Beclin-1, thereby inhibiting the initiation and completion of autophagic flux. On the other hand, impaired autophagy leads to further accumulation of dysfunctional mitochondria and toxic protein aggregates, thereby aggravating ROS generation and forming a vicious cycle (Li B. et al., 2016). Notably, interactions between oxidative stress and autophagy usually involve multiple signaling pathways. For example, ROS can activate AMPK and inhibit mTOR signaling to induce autophagy, thereby removing damaged metabolites and enhancing the self-protective capacity of cardiomyocytes (Li D. et al., 2023). However, persistently high ROS levels may overactivate autophagy or even induce autophagic cell death (Zhang J.-C. et al., 2024). Conversely, autophagy can effectively mitigate oxidative stress by selectively removing oxidatively damaged mitochondria, namely mitophagy, and oxidatively modified protein aggregates (Gouda et al., 2025). Dysregulation of this network is also reflected in HF-related pathological states. For example, dysfunction of the PINK1/Parkin signaling pathway reduces mitochondrial ROS clearance and impairs mitophagy in cardiomyocytes (Chen J. et al., 2023). Downregulation of antioxidant-related proteins such as SIRT3 weakens myocardial antioxidant defenses and indirectly affects autophagic activity (Zheng et al., 2019).

It should be noted that the relationship between ROS and autophagy is rarely linear. Depending on concentration and context, ROS can both induce and inhibit autophagy. Similar marker expression patterns, such as LC3-II and p62, may reflect opposite flux states. Many antioxidant candidates are pleiotropic and have limited bioavailability in myocardial tissue, complicating mechanistic interpretation and clinical translation. More rigorous studies should integrate pharmacokinetic data, target engagement, cell-type-specific autophagic flux assays, and disease-stage analyses.

#### 4.2.4 Autophagy and ER stress

ER stress results from excessive accumulation of unfolded or misfolded proteins within the ER and can trigger the unfolded protein response (UPR), activating three key sensor molecules, IRE1α, PERK, and ATF6, to restore protein homeostasis (Hetz, 2012). Autophagy and ER stress are two closely related cellular stress-response mechanisms. In the pathological progression of HF, they interact in complex ways and regulate disease progression (Hu et al., 2023). Because of continuous mechanical contraction, high metabolic demand, and pathological protein modifications, such as ubiquitinated protein aggregates and DAMPs released by senescent mitochondria, cardiomyocytes are particularly sensitive to ER stress. Persistent ER stress shifts the UPR from an adaptive mechanism to a proapoptotic signal. For example, sustained activation of the PERK pathway increases eIF2α phosphorylation and upregulates CHOP expression, thereby inducing cardiomyocyte apoptosis and ultimately promoting myocardial fibrosis (Zhang C. et al., 2020). Meanwhile, ER stress and autophagy are closely and bidirectionally regulated. Moderate ER stress can induce protective autophagy through PERK and IRE1 signaling pathways, promoting the removal of misfolded proteins. For example, PERK activation can upregulate ATG expression, whereas the IRE1-JNK signaling pathway can initiate autophagy by disrupting the Bcl-2/Beclin1 complex. Conversely, severe or persistent ER stress can inhibit autophagic flux and aggravate the accumulation of misfolded proteins and oxidative damage. Notably, toxic protein aggregates accumulated in HF not only trigger ER stress but also depend heavily on the autophagy-lysosome pathway for clearance. Therefore, impaired autophagy further increases ER burden and forms a vicious cycle (Cillo et al., 2025). In addition, HF-related pathological factors (such as pressure overload and ischemia/reperfusion) and genetic defects (such as mutations in sarcomeric protein genes and the lysosomal membrane protein LAMP2) can simultaneously interfere with UPR signaling and autophagic activity, highlighting the central role of this regulatory network in HF pathogenesis (Jensen et al., 2017).

Although UPR activation is often portrayed as universally harmful, early UPR responses may be compensatory. Their effects depend on factors such as the duration, intensity, and dominant arm of the specific UPR pathway. Similarly, if lysosomal capacity is limited, inducing autophagy does not necessarily enhance clearance efficiency. Excessive induction may instead increase the accumulation of intermediates. In addition, many studies use potent chemical ER stressors, such as thapsigargin and tunicamycin, which may not accurately reflect the chronic, low-grade, and multifactorial stress characteristics specific to HF. Future research urgently requires the development of models capable of more accurately capturing the temporal progression of diseases while simultaneously quantifying dynamic changes in UPR.

#### 4.2.5 Autophagy and gut microbiome

Autophagy and the gut microbiome are closely and bidirectionally regulated, and this relationship has received increasing attention in research on HF pathogenesis. Gut microbiota regulate host-cell autophagy through metabolites such as short-chain fatty acids (SCFAs), trimethylamine N-oxide (TMAO), bacterial polysaccharides, and lipopolysaccharide (LPS). For example, butyrate can promote autophagy by activating AMPK and inhibiting mTOR signaling, thereby clearing progerin (Monterrubio-Ledezma et al., 2025). In contrast, LPS produced by Gram-negative bacteria triggers inflammatory responses through the TLR4/NF-κB signaling pathway and thereby inhibits autophagy (Wang S.-T. et al., 2023). Autophagy also plays a key role in maintaining intestinal homeostasis. Autophagy-related proteins such as ATG16L1 and NOD2 in intestinal epithelial cells not only eliminate intracellular pathogens but also participate in regulating local immune responses and maintaining microbial balance (Chen S.-L. et al., 2023). In HF, dysbiosis may compromise intestinal barrier integrity, aggravate systemic low-grade inflammation, and increase circulating proinflammatory factors and metabolic toxins, such as TMAO and phenylacetylglutamine. According to the “gut-heart axis” theory, intestinal inflammatory mediators and metabolites can directly affect myocardial function through the bloodstream and participate in ventricular remodeling and HF progression (Kochkarian et al., 2025). Impaired autophagy, which may be associated with HF-related systemic metabolic disturbances or specific genetic backgrounds, aggravates cardiomyocyte stress and apoptosis by reducing the efficiency of clearing damaged mitochondria and toxic protein aggregates. In addition, patients with HF often exhibit increased intestinal permeability (“intestinal leak”), which promotes the entry of microbial products such as LPS into the circulation, activates systemic immune responses, exacerbates inflammatory myocardial injury, and ultimately accelerates the deterioration of cardiac function (Nendl et al., 2023). In animal HF models, altered microbiota structure has been observed together with decreased myocardial LC3-II levels and p62 accumulation, indicating impaired autophagic flux. Interventions such as probiotics or fecal microbiota transplantation (FMT) have been shown to improve autophagy-related markers and attenuate HF-like phenotypes (Wu S. et al., 2025). In addition, specific microbial metabolites, such as beta-hydroxybutyrate, have been shown to activate mitophagy through the mTOR pathway, further highlighting their cardioprotective potential (Chu et al., 2024).

Current discussions of the association among the microbiome, autophagy, and HF are more logically coherent than the available causal evidence can support. Microbiome findings are highly sensitive to variables such as diet, medication use, especially antibiotics and diuretics, housing conditions, and batch effects, which challenges reproducibility across cohorts. When probiotics or FMT improve cardiac function, changes in autophagic mechanisms may reflect broad immunometabolic shifts rather than direct mechanistic drivers. Finally, although the “gut-heart axis” hypothesis is compelling, the directionality, initiating nodes, and key mediators of the causal relationship between gut dysbiosis and HF remain difficult to definitively demonstrate in humans. Future work should identify specific microbial strains and metabolites, define host targets, and demonstrate flux-dependent cardioprotective effects.

#### 4.2.6 Autophagy and multiple forms of programmed cell death

Autophagy and multiple forms of programmed cell death, including necroptosis, pyroptosis, and ferroptosis, are independent yet interconnected biological processes that may jointly contribute to progressive cardiomyocyte loss and deterioration of cardiac function in HF. Impaired autophagy leads to the accumulation of toxic protein aggregates and damaged mitochondria and increases oxidative burden, thereby accelerating HF progression (Du et al., 2020). Necroptosis is a form of programmed necrosis mediated by the RIPK1/RIPK3/MLKL signaling axis and is characterized by rupture of the cell membrane and release of cellular contents, triggering a strong inflammatory response. In HF, stimuli such as pressure overload and ischemia/reperfusion can activate necroptotic pathways. Autophagy and necroptosis interact bidirectionally: on the one hand, moderate autophagy can inhibit the initiation of necroptosis by removing damaged mitochondria and ROS. On the other hand, DAMPs released during necroptosis, such as HMGB1, can inhibit autophagic flux and form a positive-feedback loop (Zhang H. et al., 2020). Pyroptosis is a lytic cell-death process dependent on inflammasome activation and caspase-1/11 mediation and is characterized by GSDMD pore formation and release of IL-1β/IL-18. In HF, excessive activation of the NLRP3 inflammasome drives cardiomyocyte pyroptosis and fibrotic remodeling. Autophagy and pyroptosis counterbalance each other, basal autophagy can directly degrade NLRP3, pro-caspase-1, and IL-1β, so as to suppress pyroptosis. However, when autophagic flux is blocked, damaged mitochondria release mtDNA, activate TLR9 and NLRP3, and promote pyroptosis. Conversely, the inflammatory environment generated during pyroptosis can inhibit TFEB nuclear translocation and impair autophagy-lysosome function (Habimana et al., 2022). Ferroptosis is an iron-dependent form of cell death driven by lipid peroxidation and characterized by inhibition of GPX4 activity, reduced antioxidant capacity, and accumulation of lipid peroxides. In HF, iron-metabolism disturbances, such as iron overload, and oxidative stress create conditions for ferroptosis and accelerate cardiomyocyte loss. In fact, autophagy and ferroptosis interact closely. Autophagy can selectively degrade ferritin, namely ferritinophagy, to release free iron. Excessive autophagy can also degrade GPX4 or induce mitochondrial dysfunction, indirectly promoting ferroptosis. Lipid peroxides and ROS generated during ferroptosis can in turn impair autophagic flux, thereby forming a vicious cycle (Yang et al., 2026). During the HF development, dysregulation of the autophagy-programmed death regulatory axis accelerates myocardial remodeling and deterioration of cardiac function. For example, mitochondrial dysfunction and oxidative stress, characteristic features of HF, are both consequences of autophagy defects and important triggers of necroptosis, pyroptosis, and ferroptosis (Wu D. et al., 2025). Therefore, restoring autophagic function and inhibiting these forms of programmed death have become key strategies for developing HF therapies. In experimental models, autophagy inducers (such as rapamycin) and specific death-pathway inhibitors (such as the necroptosis inhibitor Nec-1, the pyroptosis inhibitor Ac-YVAD-cmk, and the ferroptosis inhibitor Ferrostatin-1) have all shown cardioprotective potential.

A major challenge in this field is the difficulty of diagnosing specific forms of programmed cell death, especially pyroptosis and ferroptosis, with high specificity *in vivo*. Many proposed biomarkers overlap with conventional markers of oxidative damage or inflammation, thereby complicating the interpretation of results. Therefore, the reported benefits of death-pathway inhibitors may reflect broad antioxidant or anti-inflammatory effects rather than specific inhibition of a particular pathway. Similarly, clarifying the mechanistic links between autophagy and different forms of programmed death requires demonstration of flux dependence rather than reliance solely on changes in static markers. More rigorous studies are therefore needed to simultaneously confirm dependence on specific death pathways and the causal role of autophagy within the same experimental framework.

## 5 Regulatory mechanisms and related signaling pathways of autophagy in HF

**FIGURE 4:**
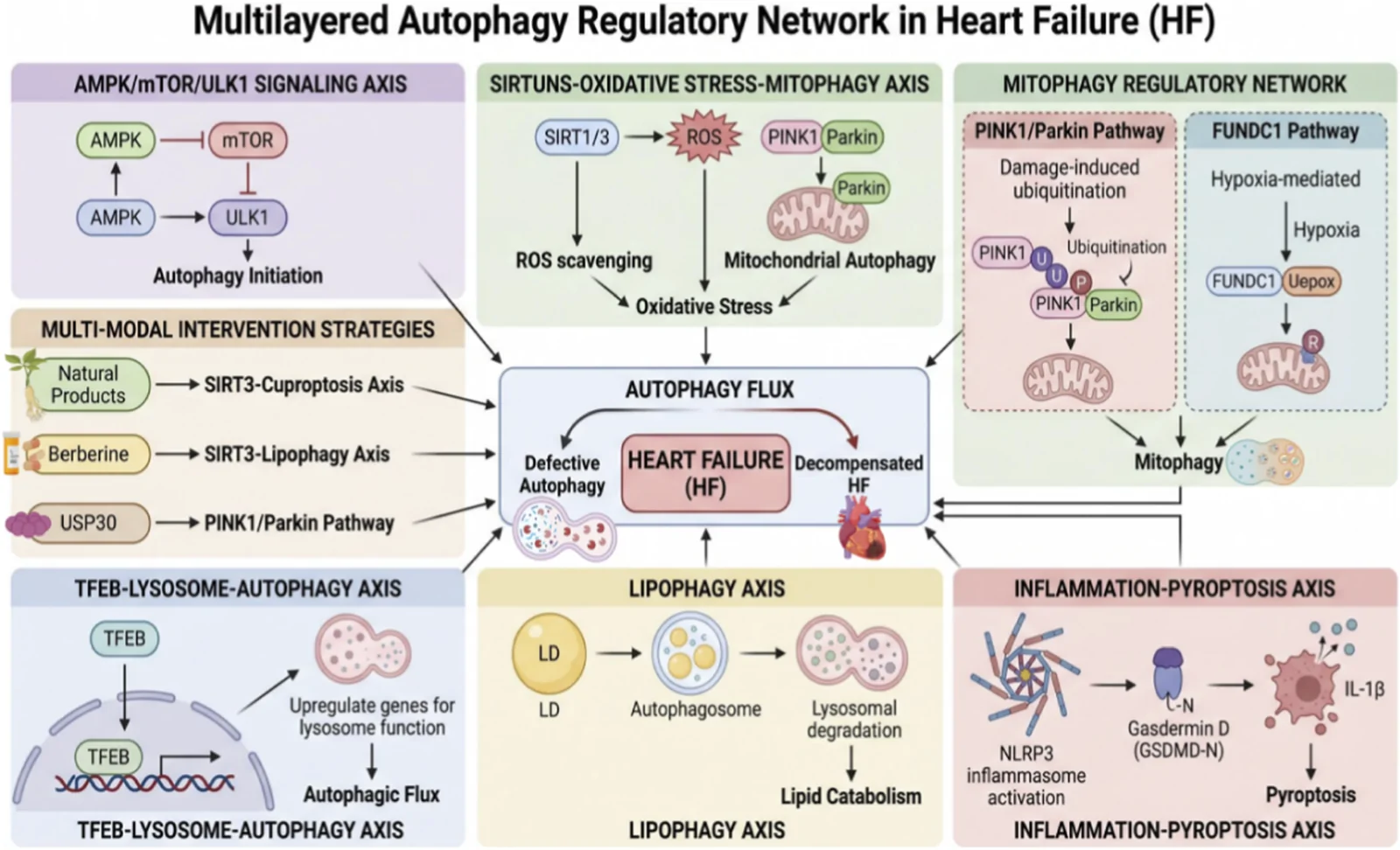
Overview of multilayered autophagy regulator network in HF. The schematic highlights key regulatory nodes modulating autophagy across cardiomyocytes, fibroblasts, and myocardial tissue, including Sirtuins-oxidative stress-mitophagy axis, AMPK/mTOR/ULK1 signaling axis, mitophagy regulatory network, TFEB-lysosome-autophagy axis, lipophagy axis, and inflammation-pyroptosis axis.

In recent years, the regulatory mechanisms of autophagy and its associated signaling pathways in HF have become a focal point of research. Current evidence indicates that these regulatory mechanisms and signaling pathways collectively form a complex network with multiple layers, nodes, and substrates. From classic energy-sensing nodes to substrate-specific autophagy, and from epigenetic regulation to crosstalk with cell death pathways, multiple signaling pathways interact and regulate one another. Among them, the interconnections and feedback regulation among the Sirtuins-oxidative stress-mitophagy axis, the AMPK/mTOR/ULK1 signaling axis, the mitophagy regulatory network, including the PINK1/Parkin and FUNDC1 pathways, the TFEB-lysosome-autophagy regulatory axis, lipophagy, and the inflammation-pyroptosis axis jointly determine the ultimate autophagic status during HF progression. In addition, multiple multimodal intervention strategies are continuously deepening our understanding of HF pathogenesis, for example, natural products regulating autophagy through the SIRT3-cuproptosis axis, berberine through the SIRT3-lipophagy axis, and USP30 through the PINK1/Parkin pathway. These studies also suggest multiple directions for drug development and clinical intervention (Figure 4).

### 5.1 Sirtuins-oxidative stress-mitophagy axis

Sirtuins are a highly conserved class of NAD^+^-dependent deacetylases that act as core sensors of energy metabolism, oxidative stress, and cellular stress responses, and they participate in multiple biological processes by regulating protein deacetylation. In mammals, the Sirtuin family contains seven members (SIRT1-SIRT7), among which SIRT1, SIRT6, and SIRT7 are mainly localized in the nucleus, SIRT3, SIRT4, and SIRT5 are distributed in mitochondria, and SIRT2 is primarily located in the cytoplasm. Notably, SIRT1, SIRT3, and SIRT6 play core positive regulatory roles in autophagy and mitochondrial quality control. Mechanistic studies show that Sirtuin activity is precisely regulated by upstream signals. Increased NAD^+^ levels activate their catalytic activity, whereas oxidative stress and inflammatory environments inhibit their function by depleting NAD^+^ (Wang Y.-Q. et al., 2023). First, SIRT1 is localized in the nucleus and mainly upregulates the expression of ATGs, such as LC3, BNIP3, ATG5, and ATG7, and lysosomal biogenesis-related genes by deacetylating FOXO1/3 transcription factors. It also enhances mitochondrial biogenesis and oxidative metabolism by deacetylating PGC-1α, thereby promoting autophagy and improving myocardial energy homeostasis. However, during HF, decreased NAD^+^ levels and increased oxidative stress inhibit SIRT1 activity, leading to impaired autophagic flux, toxic protein accumulation, and aggravated cardiomyocyte apoptosis (Ding et al., 2024). Second, SIRT3 is localized in mitochondria and is a core regulator of mitophagy and antioxidant defense. Its mechanisms operate in two ways: on the one hand, it initiates ubiquitin-dependent mitophagy by deacetylating PINK1/Parkin (Liu P. et al., 2025). On the other hand, it enhances reactive oxygen species (ROS) scavenging by deacetylating MnSOD and improves the binding efficiency between LC3 and damaged mitochondria (Sultana et al., 2016). During the HF progression, downregulation of SIRT3 impairs mitophagy, aggravates ROS accumulation and mitochondrial injury, and ultimately forms a vicious cycle (Hu et al., 2022). In addition, SIRT6 is localized in the nucleus and regulates autophagy through AMPK/mTOR- and FOXO3-dependent pathways, activates autophagy initiators such as ATG5 and ATG7, and modulates HIF-1α/NF-κB signaling to reduce oxidative stress and cardiac fibrosis. In diabetic cardiomyopathy, reduced SIRT6 expression is closely associated with impaired autophagic flux, lipid accumulation, and aggravated inflammation (Zhan et al., 2025). Taken together, the coordinated regulation of SIRT1, SIRT3, and SIRT6 constitutes the core framework of the “Sirtuins-oxidative stress-mitophagy axis” in HF. SIRT1 mainly regulates transcriptional expression of nuclear autophagy genes. SIRT3 focuses on the initiation and execution of mitophagy and antioxidant defense. And SIRT6 links autophagy regulation through energy-sensing pathways. During HF, decreased NAD^+^ levels, enhanced oxidative stress, and reduced Sirtuin activity jointly lead to impaired autophagic flux and collapse of the mitochondrial quality-control system. Meanwhile, abnormally accumulated mitochondria release ROS, further consume NAD^+^, and inhibit Sirtuin activity, thereby forming a positive-feedback loop that accelerates cardiomyocyte loss and deterioration of cardiac function (Krekora et al., 2025).

Notably, the relationship between Sirtuins and oxidative stress in autophagy regulation is markedly concentration- and context-dependent. On the one hand, moderate enhancement of Sirtuin activity can restore autophagic flux, remove damaged mitochondria, reduce oxidative stress, and exert cardioprotective effects. On the other hand, excessive Sirtuin activation may cause overconsumption of NAD^+^ or excessive mitophagy, thereby weakening the energy-supplying capacity of cardiomyocytes. In advanced HF, reduced Sirtuin activity may represent a myocardial compensatory response to energy deprivation. Therefore, exogenous strategies, such as NAD^+^ precursors, Sirtuin activators, or natural products, that precisely restore Sirtuin activity to normal levels or induce moderate activation may be more therapeutically appropriate than overexpression or complete inhibition. Collectively, SIRT1, SIRT3, and SIRT6 coordinately regulate autophagy and mitochondrial quality through deacetylation and downstream signaling pathways. These downstream pathways include the transcription factors FOXO1/3 and PGC-1α, the core mitophagy proteins PINK1/Parkin, the energy-sensing AMPK/mTOR pathway, and the autophagy-lysosome regulator TFEB. Current evidence suggests that precise pharmacological modulation of Sirtuin activity to restore autophagic function may be a promising strategy for HF treatment (Zhu et al., 2025). However, to avoid bioenergetic depletion caused by excessive mitophagy or interference with the heart’s natural compensatory mechanisms, the optimal intensity, timing, and target selectivity of Sirtuin interventions must still be defined, this remains a key challenge in translational medicine.

### 5.2 AMPK/mTOR/ULK1 signaling axis

AMPK is a highly conserved serine/threonine protein kinase in eukaryotes that functions as a core energy sensor by detecting changes in the AMP/ATP ratio. Under energy-deprived conditions such as myocardial ischemia, pressure overload, or metabolic stress, AMP binds to the regulatory gamma subunit and induces conformational changes, prompting upstream kinases, such as LKB1 and CaMKKβ, to phosphorylate Thr172 on the alpha subunit and thereby activate AMPK (Lin and Hardie, 2018). In autophagy, AMPK exerts key positive regulatory effects through multiple mechanisms. It directly phosphorylates ULK1 at Ser317 and Ser777 to initiate autophagy. It inhibits mTORC1 by phosphorylating TSC2 and Raptor, thereby relieving mTORC1-mediated inhibition of ULK1. Moreover, AMPK regulates the expression of ATGs by targeting transcription factors such as FOXO3 and PGC-1α, and promotes VPS34 complex activity and PI3P production by modifying Beclin1 (Li and Chen, 2019). Thus, the AMPK/mTOR/ULK1 signaling axis constitutes the core regulatory network for autophagy initiation under energy stress. Given its central role in energy sensing and metabolic regulation, AMPK has attracted increasing attention in HF research. In metabolic heart disease, AMPK activation improves myocardial energy metabolism, enhances insulin sensitivity, and reduces lipotoxicity (Hardie, 2008). In ischemic heart disease, AMPK exerts acute cardioprotective effects by inducing autophagy to remove damaged mitochondria (Qi and Young, 2015). However, in pressure overload-induced chronic heart failure (CHF), AMPK exhibits context-dependent dual functions: moderate activation helps maintain cardiomyocyte homeostasis, whereas chronic overactivation may accelerate cardiomyocyte loss and ventricular remodeling by promoting NADPH oxidase activation and aggravating oxidative stress (Li et al., 2018).

Recent HF studies have further confirmed the cardioprotective potential of the AMPK/mTORC1/ULK1 signaling pathway. Certain drugs or natural products can improve cardiac function and delay HF progression by increasing the p-AMPK/AMPK ratio, enhancing autophagy-lysosome function, and reducing the accumulation of abnormal protein aggregates, such as ubiquitinated proteins (Liu C. et al., 2023). Nevertheless, although existing evidence supports a protective role for AMPK in HF, its regulation remains highly complex, mainly in the following respects. First, AMPK has dual effects: acute activation can promote cardiomyocyte survival through autophagy, whereas chronic overactivation may induce cardiomyocyte death through excessive ROS generation and mitochondrial dysfunction. Second, its effects are cell-type specific: it may protect cardiomyocytes but promote fibrotic or inflammatory responses in cardiac fibroblasts or macrophages. Third, the interactions between AMPK and HF-related pathways, such as Sirtuins, PINK1/Parkin, and the NLRP3 inflammasome, remain unclear, and whether AMPK dysfunction is a cause or consequence of HF has not been determined. Fourth, most studies are based on acute intervention models, such as ischemia/reperfusion, and the long-term effects of AMPK regulation in CHF models remain insufficiently evaluated. Finally, the threshold between protective and pathogenic autophagy remains undefined, and systemic AMPK activation may cause peripheral metabolic disturbances, such as hypoglycemia and lactic acidosis. Therefore, although the AMPK/mTOR/ULK1 signaling axis remains a promising therapeutic target, future strategies should focus on stage-specific temporal regulation, cell-type-specific targeting, and network-level coordination rather than simple unidirectional activation.

### 5.3 Mitophagy regulatory network

The mitophagy regulatory network is a core mechanism for removing damaged mitochondria and maintaining myocardial energy homeostasis. This network mainly consists of two pathways: the ubiquitin-dependent pathway mediated by PINK1/Parkin and the receptor-mediated non-ubiquitin-dependent pathway involving BNIP3/NIX and FUNDC1. In the ubiquitin-dependent pathway, PINK1 is stabilized on the outer membrane of depolarized mitochondria, phosphorylates ubiquitin, and recruits Parkin. Damaged mitochondria are then marked with ubiquitin chains, recognized by autophagy receptors such as p62, OPTN, and NDP52, linked to LC3, and ultimately engulfed by autophagosomes (Zhao et al., 2024). The receptor-mediated pathway removes mitochondria through direct interactions of BNIP3 or FUNDC1 with LC3 (Lampert et al., 2019). These two pathways jointly promote phagophore expansion and closure through phosphorylation and lipid signaling. This network activity is tightly regulated by multiple mechanisms: (1) deubiquitinating enzymes, such as USP30 and USP35, inhibit mitophagy by removing Parkin-mediated ubiquitin chains, whereas kinases, such as PINK1 and TBK1, enhance its activity. (2) Among upstream pathways, AMPK activates mitophagy by phosphorylating ULK1 and promoting Parkin translocation, whereas mTOR suppresses autophagy initiation under nutrient-rich conditions. (3) SIRT3 enhances PINK1 and Parkin function through deacetylation, whereas oxidative stress consumes NAD^+^ and inhibits SIRT3, thereby weakening mitophagy (Onishi et al., 2021). Notably, dysregulation of this network is closely associated with HF. Loss or dysfunction of PINK1 or Parkin can lead to the accumulation of damaged mitochondria, excessive ROS generation, and cardiomyocyte apoptosis, thereby accelerating ventricular remodeling. Abnormal BNIP3 expression in HF also affects mitochondrial quality control (Liu Y. et al., 2023). In pressure overload or ischemia/reperfusion models, impaired mitophagy prevents the removal of toxic protein aggregates and oxidatively damaged mitochondria, thus accelerating deterioration of cardiac function (Li X.-T. et al., 2025).

Although the association between the mitophagy regulatory network and HF has been relatively well established, several key issues remain unresolved. First, it is unclear whether dysfunction of this network is a direct cause of HF pathology or a secondary consequence of disordered myocardial energy metabolism. Although reduced PINK1 levels are associated with mitochondrial injury, a causal relationship has not been established. Second, mitophagic behavior is context-dependent, complicating therapeutic targeting: moderate enhancement can remove damaged mitochondria, whereas excessive activation may lead to excessive elimination of healthy mitochondria and weaken myocardial energy supply. In addition, mitophagy intersects with apoptotic pathways, for example Parkin can inhibit apoptosis, so modulating its activity may have unexpected effects on cell survival. Third, different receptor pathways, such as the PINK1/Parkin and BNIP3/FUNDC1 pathways, may play different roles in cardiomyocytes, fibroblasts, and immune cells, but cell-type-specific regulatory mechanisms remain unclear. Fourth, current evidence is mostly derived from acute cellular or animal models, such as ischemia/reperfusion and drug-induced models, and validation in myocardial tissues from patients with CHF or in long-term *in vivo* systems remains very limited. Fifth, interactions between the mitophagy network and HF-related signaling pathways, such as Sirtuins, AMPK, and the NLRP3 inflammasome, have only begun to be clarified, and the network-level cascade effects within multipathway crosstalk remain undefined. Finally, the optimal therapeutic window remains unclear: insufficient activity fails to clear damaged mitochondria, whereas excessive enhancement may disturb mitochondrial dynamics, including fusion and fission, and induce metabolic crisis. Therefore, although targeting the mitophagy regulatory network is promising, successful strategies must achieve cell-type specificity and temporal precision to restore mitochondrial quality control without disrupting overall myocardial homeostasis.

### 5.4 TFEB-lysosome-autophagy regulatory axis

When cardiomyocytes are exposed to internal or external stimuli, such as pressure overload, ischemia/hypoxia, or oxidative stress, a series of conserved signaling pathways is activated to restore cellular homeostasis. Among them, TFEB is a core transcription factor that regulates lysosomal biogenesis and autophagy, and its activity is precisely controlled by multiple signals, including mTORC1, lysosomal status, and oxidative stress (Wen et al., 2023; Yan et al., 2024). Under basal conditions, mTORC1 phosphorylates TFEB and retains it in the cytoplasm. When cells experience nutrient deprivation or lysosomal dysfunction, mTORC1 activity decreases, TFEB becomes dephosphorylated and translocates to the nucleus, where it activates the expression of ATGs, such as LC3, ATG5, and p62, and lysosome-related genes, such as LAMP1, CTSD, and ATP6V1H (Sardiello et al., 2009; Settembre et al., 2011). In addition, oxidative stress can indirectly promote TFEB nuclear translocation by activating AMPK and inhibiting mTORC1, whereas lysosomal stress directly dephosphorylates TFEB through calcium release and calcineurin activation, thereby initiating the autophagy-lysosome repair response (Ran et al., 2025). Under hypoxic conditions, HIF-1α does not directly regulate TFEB but can upregulate mitophagy receptors such as BNIP3, thereby cooperating with TFEB to enhance the removal of damaged mitochondria (Zhang Y. et al., 2019). Notably, extensive crosstalk exists among these pathways: oxidative stress and lysosomal stress often occur simultaneously, TFEB and the Nrf2 signaling pathway synergistically enhance antioxidant defense and autophagy-lysosome function, and ROS induced by chronic hypoxia further aggravate lysosomal dysfunction and inhibit TFEB activity (Popov et al., 2023).

In HF, altered activity of the TFEB-lysosome-autophagy regulatory axis is closely associated with cardiomyocyte loss and ventricular remodeling. Moderate TFEB activation helps restore autophagic flux and remove toxic protein aggregates and damaged mitochondria, thereby delaying HF progression. Deeper investigation, however, indicates that TFEB regulation under this pathological condition has a typical “double-edged sword” property: physiological activation promotes cardiomyocyte survival, whereas sustained lysosomal stress and oxidative stress in CHF may lead to TFEB dysfunction, such as impaired nuclear translocation and reduced transcriptional activity, causing collapse of the autophagy-lysosome system and aggravating proteotoxic stress and cell death. Therefore, although targeting the TFEB axis has clear therapeutic potential, its role in HF is context-dependent and often bidirectional. It must be carefully regulated to restore lysosomal homeostasis and autophagic balance while avoiding increased metabolic burden on cardiomyocytes or interference with cardiac compensatory mechanisms.

### 5.5 Lipophagy and myocardial lipotoxicity

Lipophagy is a form of selective autophagy that is primarily responsible for degrading intracellular lipid droplets and plays a key role in maintaining myocardial lipid metabolism and energy homeostasis. Dysfunction of this pathway is closely associated with lipotoxicity in obesity, diabetes-related cardiomyopathy, and HF, highlighting its important pathological significance (Cheong et al., 2025; Ke et al., 2024; Leggat et al., 2021). Under basal conditions, lipid droplets in cardiomyocytes are coated with lipid droplet-associated proteins, such as PLIN1, PLIN2, and PLIN5, which prevent autophagy receptors from binding to the lipid droplet surface and thereby maintain stable storage of lipid droplets. When cells are exposed to a high-fat environment, oxidative stress, or energy stress, surface proteins of lipid droplets undergo phosphorylation or ubiquitination modifications, leading to their dissociation or degradation and subsequently exposing the lipid droplet core. Meanwhile, autophagy receptors, such as p62, NDP52, and OPTN, are recruited around lipid droplets and bind LC3 through their LC3-interacting regions, thereby initiating lipophagy (Liu and Zhao, 2025). In addition, AMPK activation promotes lipid droplet breakdown, whereas mTOR inhibition enhances overall autophagic flux and indirectly promotes lipophagy (Zhou et al., 2025). Beyond regulating lipid droplet degradation, lipophagy also affects fatty acid oxidation, insulin sensitivity, and inflammatory responses, further underscoring its broad role in myocardial metabolic regulation (Wu Y. et al., 2026).

In HF, lipophagy dysfunction impairs lipid droplet clearance, leading to abnormal lipid accumulation in cardiomyocytes, buildup of lipotoxic intermediates such as ceramides and diacylglycerols, and aggravated oxidative stress, which in turn induces cardiomyocyte apoptosis and ventricular remodeling. Recent studies using animal models of diabetic cardiomyopathy have confirmed that defects in lipophagy-related genes, such as ATG7 and p62, result in myocardial lipid accumulation and progressive deterioration of cardiac function, closely recapitulating the core features of lipotoxic cardiomyopathy (Tong et al., 2019). Further work, however, has revealed important distinctions. Although lipophagy is undoubtedly a central mechanism in metabolic cardiomyopathy, its role in non-metabolic HF, such as pressure overload-induced HF, remains unclear. Existing evidence indicates that lipophagy deficiency alone may be insufficient to induce HF and usually requires additional stressors, such as oxidative stress or chronic inflammation, to manifest a pathological phenotype (Chai et al., 2026). In addition, the specific hierarchy of different autophagy receptors in lipophagy, and whether alternative lipid-clearance pathways, such as cytosolic lipases, can compensate for lipophagy loss in cardiomyocytes, remain controversial. From a therapeutic perspective, enhancing lipophagy has considerable potential but must be approached cautiously. Excessive lipophagy may overconsume lipid droplet reserves in cardiomyocytes, leading to insufficient energy supply, because the myocardium relies heavily on fatty acid oxidation, and may consequently aggravate metabolic crisis. Therefore, although targeting the lipophagy pathway is promising, successful therapeutic strategies will likely require precise regulation to restore lipid metabolic balance rather than merely increasing lipophagic flux.

### 5.6 Autophagy-inflammasome-pyroptosis axis

The autophagy-inflammasome-pyroptosis axis is a core functional network that regulates myocardial inflammatory responses and cell death in HF. Within this axis, autophagy suppresses excessive activation of the NLRP3 inflammasome by removing damaged mitochondria and damage-associated molecular patterns (DAMPs), whereas pyroptosis is a lytic form of cell death triggered by inflammasome activation and characterized by perforation of GSDMD and massive release of IL-1β and IL-18 (Chiu et al., 2017). Autophagic regulation of the inflammasome involves multiple mechanisms. For example, autophagy can selectively degrade key metabolites such as NLRP3, ASC, pro-caspase-1, and mature IL-1β. Mitophagy removes damaged mitochondria that release mtDNA, thereby blocking NLRP3 activation signals, and autophagy can also influence inflammasome transcription by modulating the NF-κB signaling pathway (Su et al., 2025). When autophagy is impaired, accumulated ROS and mtDNA strongly activate the NLRP3 inflammasome, which promotes caspase-1-mediated cleavage of GSDMD, pore formation, and pyroptosis. Notably, IL-1β and IL-18 released during pyroptosis can further inhibit autophagic flux, for example by downregulating TFEB activity, thereby establishing a vicious cycle of positive feedback (Oka et al., 2012). The integrity of this axis is essential for maintaining cardiomyocyte homeostasis. Its dysfunction has been shown to be closely associated with ischemic heart disease, diabetic cardiomyopathy, and pressure overload-induced HF (Yu et al., 2024; Lu et al., 2025; Zhou Y. et al., 2024).

In HF, increasing evidence indicates a close association between impaired autophagy, excessive NLRP3 inflammasome activation, and cardiomyocyte pyroptosis. Reduced expression of ATGs, such as ATG5 and ATG7, or lysosomal dysfunction weakens inflammasome clearance, resulting in sustained NLRP3 activation, increased GSDMD cleavage, and cardiomyocyte pyroptosis, which further exacerbate inflammatory infiltration and ventricular remodeling. HF-related genetic variants, such as gain-of-function mutations in NLRP3, or environmental factors, such as pressure overload and ischemia/reperfusion injury, can accelerate HF progression by disrupting the autophagy-inflammasome balance (Hammami et al., 2025). However, a more detailed evaluation reveals substantial complexity: although autophagy defects and inflammasome activation often coexist in HF models, whether impaired autophagy is a direct trigger of pyroptosis or a secondary manifestation of systemic metabolic and inflammatory disorders remains unclear. In addition, the relationship between inflammasome activity and pyroptosis severity is nonlinear. Elevated NLRP3 levels may arise from transcriptional upregulation or impaired autophagic degradation, and interpretation therefore requires caution. No consensus has yet been reached regarding the pathophysiological significance of partial autophagy dysfunction, rather than complete loss, in CHF. Meanwhile, compensatory mechanisms involving other immunoregulatory pathways, such as NF-κB and MAPK, may partially offset the consequences of autophagy defects, further complicating therapeutic strategies. Therefore, although the autophagy-inflammasome-pyroptosis axis remains an attractive biomarker and potential therapeutic target, interventions must fully consider its fundamental role in immune defense and the infection risk associated with excessive inflammasome inhibition. Successful intervention should focus on fine-tuned regulation, such as enhancing autophagy without completely blocking pyroptosis or targeting upstream DAMPs, rather than broad activation or inhibition of a single link. At the same time, the dynamic balance between autophagic flux and inflammation must be continuously monitored across all stages of HF pathology.

## 6 Medicinal plants and their active metabolites delay the HF progression by targeting autophagic flux

**FIGURE 5:**
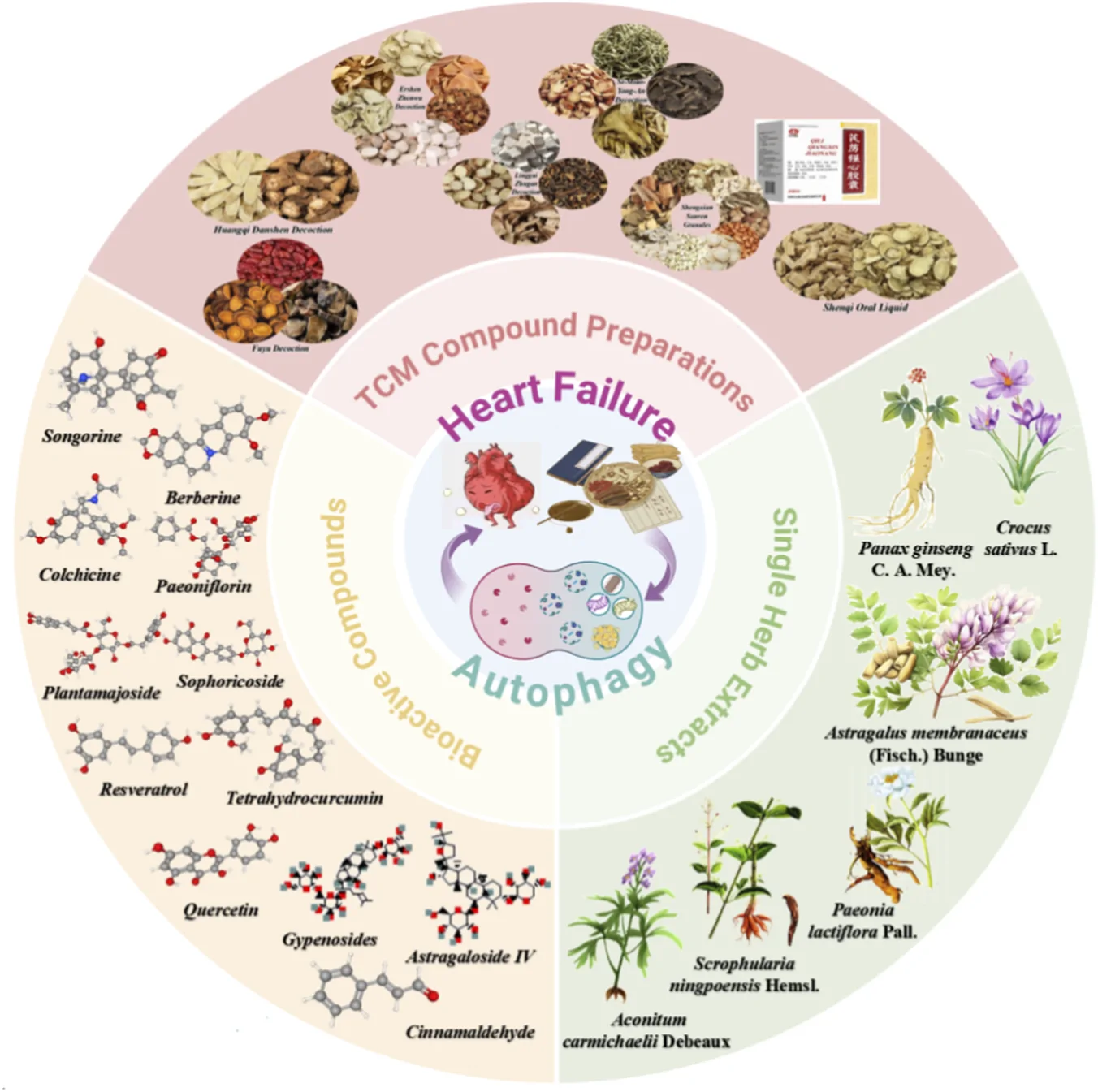
Intervention strategies to delay the progression of HF by targeting autophagic flux, including the TCM compound preparations, single-botanical drug extracts, and bioactive metabolites.

**FIGURE 6:**
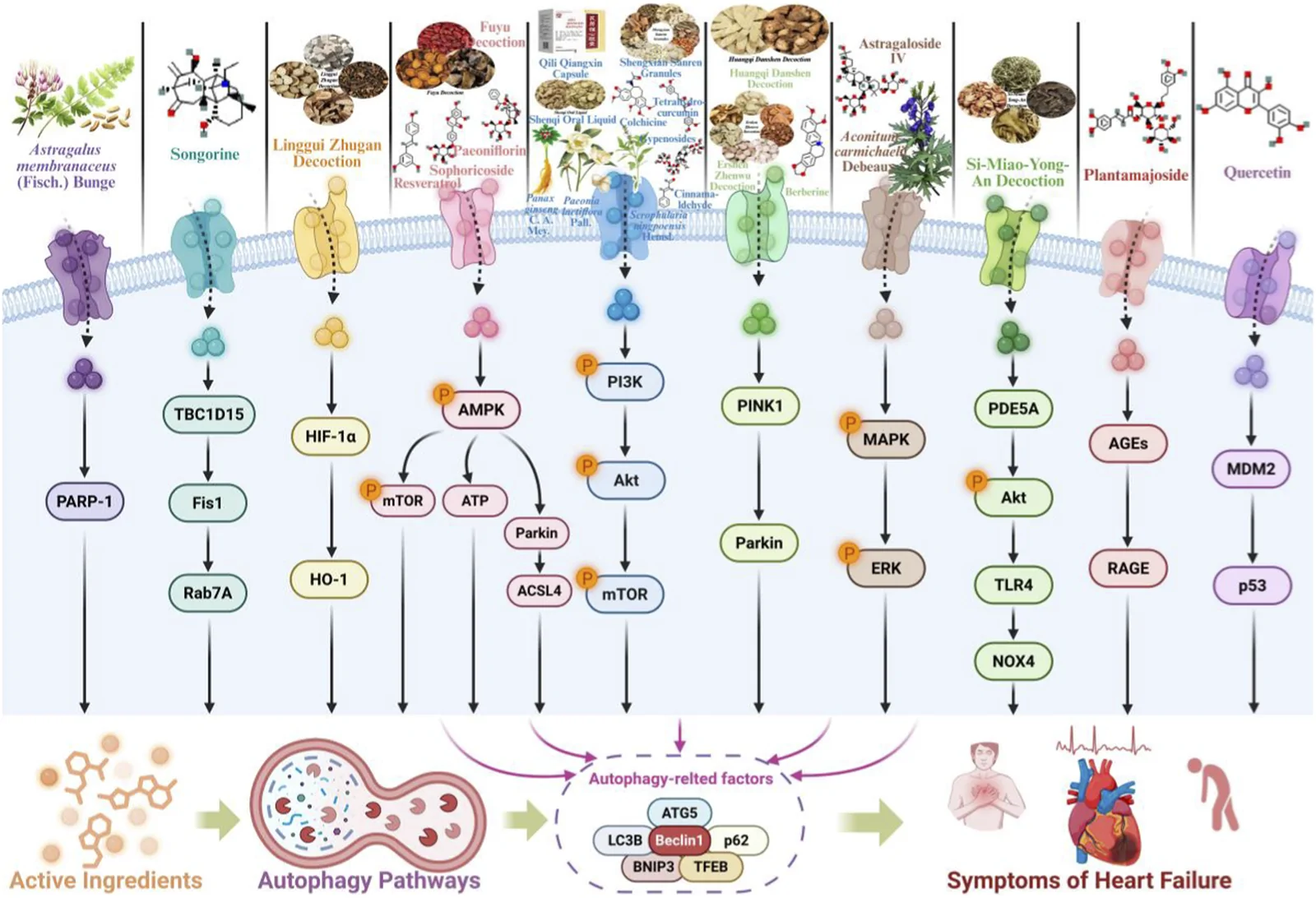
Medicinal plants (including the herbal formulation, single herbal extracts, and active metabolites) of TCM act on autophagy-related pathways to delay the progression of HF.

Recent studies indicate that medicinal plants are characterized by “multi-metabolite, multi-target, and multi-pathway” actions, which not only align with the holistic therapeutic principles of TCM but also closely match the complex pathological mechanisms of HF (Shao-Mei et al., 2022). Modern pharmacological research has confirmed that these botanical drugs possess multiple biological activities, including antioxidant, anti-inflammatory, anti-apoptotic, and cardioprotective effects, with relatively low hepatotoxicity and nephrotoxicity (Suo et al., 2024). Their active metabolites, such as alkaloids, phenols, flavonoids, saponins, terpenoids, and anthraquinones, can effectively regulate autophagic flux in patients with HF through key signaling pathways including PI3K/Akt/mTOR, AMPK/mTOR, PINK1/Parkin, SIRT1, SIRT3, and TFEB (Figures 5, 6; Tables 1–3). These findings provide a scientific basis for the application of medicinal plants in HF treatment and highlight their potential in regulating autophagy and maintaining myocardial energy homeostasis and cellular quality control (Tables 4–6). Accordingly, herbal regulation of autophagy is emerging as a promising strategy for the treatment of HF.

**TABLE 1:** TCM decoction delays the progression of HF by targeting autophagic flux.

Chinese herbal compound preparations	Traditional uses for cardiovascular disease	Dosage/route/time	Autophagy modulators/positive controls	Related targets	Pharmacological mechanism	*In vitro*/*vivo* model	References
Huangqi Danshen Decoction	HF	50, 100, 150, 200, 250, 300 μg/mL, medicated culture medium for 24 h; 4.69, 9.38 g/kg/day, intragastric administration for 8 weeks	Perindopril, miR-27a-3p mimics	TSG101, CD63, TNF-α, IL-1β, IL-6, IL-18, COXIV, Prkaa2, Prkn, Map1lc3b, Sqstm1, p62, LC3B	Activating mitophagy through the AMPK/PINK1/Parkin signaling pathway	Ang II-induced H9C2 cells injury, TCA surgery-induced male SD rats model	Chen Z. et al. (2024)
Fuyu Decoction	HF	5.0 g/kg/day, intragastric administration for 8 weeks	AMPK agonist EX229	LC3B, Beclin1, p62	Inhibiting AMPK/mTOR signaling pathway-mediated autophagy	Ligation of LAD-induced male Wistar rats model	Ma et al. (2023)
Ershen Zhenwu Decoction	HF (Cheng et al., 2025), DOX-induced cardiotoxicity (Yue et al., 2025)	5%, 10%, 15%, 20%, drug-containing serum for 24 h	Valsartan, 3-MA	LC3B, Beclin1, p62	Modulating the PINK1/Parkin signaling pathway to inhibit mitophagy	Thyroidectomy and DOX injection-induced male SD rats model	Wang X. et al. (2026)
Linggui Zhugan Decoction	HF (Yang et al., 2023), DOX-induced cardiotoxicity (Kong et al., 2025), coronary heart disease (Liu L. et al., 2022), myocardial infarction (Wang et al., 2020)	2.34, 4.68, 9.36 g/kg/day, intragastric administration for 6 weeks	Captopril	ATG5, LC3B, Beclin1, p62	Enhancing autophagy through the upregulation of the HIF-1α/HO-1 signaling pathway	Ligation of LAD-induced male C57BL/6 J mice model	Ren et al. (2024)
Si-Miao-Yong-An Decoction	HF (Zhao et al., 2020), diabetic cardiomyopathy (Li L. et al., 2025), acute coronary syndrome (Zhao et al., 2025), coronary heart disease (Zhang J.-M. et al., 2024), myocardial infarction (Cui et al., 2021)	10, 30 μM, medicated culture medium for 48 h; 15, 30, 100 mg/kg/day, intragastric administration for 4 weeks	SC79, MK2206, MHY1485, rapamycin, 3-MA, LYN1604, SBI-0206965, si-PDE5A, si-TLR4, digoxin	p-mTOR, ULK1, Bcl-2, LC3B, Beclin1, p62	Attenuating autophagy *via* the PDE5A/Akt and TLR4/NOX4 signaling pathways	ISO-induced H9C2 injury and male SD rats model	Liao et al. (2022)
Qili Qiangxin capsule	HF (Li et al., 2013), dilated cardiomyopathy (Wei et al., 2022), pulmonary arterial hypertension (Han X. et al., 2022), myocardial infarction (Sun et al., 2024)	0.5, 1, 1.25, 2.5, 3.75, 5, 10, 20, 50, 100 μg/mL, medicated culture medium for 24 h; 0.234, 0.468, 0.936 g/kg/day, intragastric administration for 7 days	TMZ, LY294002	LC3B, Beclin1, p62	Suppressing autophagy *via* the PI3K/Akt/FoxO3 signaling pathway	H/R-induced H9C2 cells injury, ligation of LAD-induced male C57BL/6 J mice model	Wu N. et al. (2025)
Shengxian Sanren granules	HF	6.75, 13.5, 27 g/kg/day, intragastric administration for 4 weeks	Furosemide	LC3B, p62, Beclin1	Activating autophagy *via* the PI3K/Akt/mTOR signaling pathway	DOX injection-induced male SD rats model	Dai et al. (2025)
Shenqi oral liquid	HF	2.5%, 5%, 10% drug-containing serum for 24 h; 0.6, 1.2 g/kg/day, intragastric administration for 4 weeks	Rapamycin	p62, LC3B, ULK1, Beclin1, p-Drp1, MFF, MFN1, MFN2, OPA1	Inhibiting excessive autophagy by restraining the PI3K/Akt/mTOR and AMPK signaling pathways	DOX-induced H9C2 injury and male C57BL/6 J mice models	Xie et al. (2026)

TCM, traditional chinese medicine; HF, heart failure; TCA, transverse aortic constriction; SD, Sprague-Dawley; LAD, left anterior descending coronary artery, 3-MA, 3-Methyladenine; DOX, doxorubicin, H/R, Hypoxia/reoxygenation, ISO, isoproterenol.

**TABLE 2:** Extract of a single botanical drug delays the progression of HF by targeting autophagic flux.

Single-flavored TCM extract	Traditional uses for cardiovascular disease	Dosage/Route/Time	Autophagy modulators/Positive controls	Related targets	Pharmacological mechanism	*In vitro*/*vivo* model	References
*Panax ginseng* C. A. Mey. extract	HF (Xie et al., 2024), atherosclerosis (Zhou Z. et al., 2024), myocardial infarction (Xu et al., 2025)	1.3, 2.6 g/kg/day, intragastric administration for 4 weeks	Met	LC3B, Beclin1, ATG5, p62	Inhibition of autophagy mediated by the activation of the PI3K/Akt/mTOR signaling pathway	Ligation of LAD-induced male C57BL/6 J mice model	Zi-Chang et al. (2024)
*Crocus sativus* L. extract	HF, myocardial infarction (Fang et al., 2022), coronary heart disease (Meng et al., 2022), atherosclerosis (Yang R. et al., 2024), hypertension (Hu et al., 2026), barth syndrome cardiomyopathy (Zhu et al., 2024 )	10, 20, 50, 100 μg/mL, medicated culture medium for 24 h	Rapamycin, 3-MA	LC3B, BAD, Bax	Activating autophagy through the regulation of autophagical factors	Ang II-induced H9C2 cells injury model	Jia et al. (2015)
*Astragalus membranaceus* (Fisch.) Bunge injection	HF (Chen Q. et al., 2024), viral myocarditis (Zheng et al., 2020), diabetic cardiomyopathy (Sun et al., 2023), hypertrophic cardiomyopathy (Qin et al., 2025), radiation-induced heart disease (Jiang B. et al., 2025), myocardial infarction (Ma et al., 2013), coronary heart disease (Liu Z. et al., 2026)	0.25%, 0.5%, 1%, drug-containing serum for 24 h; 2.5, 5.5 mL/kg/day, intraperitoneal injections for 4 weeks	SC79, GSK-690693, ACEI	LC3B, TOMM20, p62, BNIP3	Inducing mitophagy through the inhibition of Akt/mTOR signaling pathway	PE-induced neonatal mouse cardiomyocyte, TAC-induced male C57BL/6N mice model	Li S. et al. (2025)
Total glucosides of *Paeonia lactiflora* Pall	HF	10 μg/mL, medicated culture medium for 12 h; 200, 400 mg/kg/day, intragastric administration for 12 weeks	si-PARP-1, OE-PARP-1, pcDNA3.1-PARP-1, captopril	NF-κB, cleaved Caspase-3, LC3B, TNF-α, IL-6	Inhibiting autophagy through the suppression of PARP-1	DOX-induced H9C2 cells injury, ligation of LAD-induced male Wistar rats model	Wei et al. (2024)
Total saponin of *Scrophularia ningpoensis* Hemsl	HF	30, 60, 120 mg/kg/day, intragastric administration for 4 weeks	Shakubar, trivalsartan	LC3B, p62, Beclin1	Inhibiting autophagy through the activation of Akt/mTOR signaling pathway	DOX injection-induced male Wistar rats model	Dai et al. (2023)
*Aconitum carmichaelii* Debeaux extract	HF (Xing et al., 2023), myocardial infarction (Wu et al., 2019), coronary heart disease (Li et al., 2020), atherosclerosis (Li et al., 2020), low blood pressure (Zhou et al., 2015)	0.018, 0.037, 0.074 μM, medicated culture medium for 24 h; 0.02, 0.06 mg/kg/day, intragastric administration for 10 days	NMS 783, ML-240, sacubitril valsartan sodium tablets	SERCA2, LIPT1, HSP60, BNIP3, LC3B, ATP5A, UQCRC2, MTCO1, SDHB, NDUFB8	Inhibiting autophagy and preventing remodeling of mitochondria to inhibit mitophagy by the inhibition of MAPK-ERK pathways	DMEM containing 2% sodium barbital (w/v)-induced H9C2 cells for 135 min, DOX injection-induced male C57BL/6 J mice model	Liu Y. et al. (2026)

TCM, traditional chinese medicine; HF, heart failure, Met, Metoprolol, LAD, left anterior descending coronary artery, 3-MA, 3-Methyladenine; PE, phenylephrine; TCA, transverse aortic constriction; DOX, doxorubicin.

**TABLE 3:** TCM active metabolite delays the progression of HF by targeting autophagic flux.

Category	Chinese herbal active metabolites	Medicinal plant	Traditional uses for cardiovascular disease	Dosage/Route/Time	Autophagy modulators/Positive controls	Related targets	Pharmacological mechanism	*In vitro*/*vivo* model	References
Alkaloids	Songorine	*Aconitum carmichaelii* Debeaux	HF, DOX-induced cardiomyopathy (Ding et al., 2023), septic cardiomyopathy (Chen M. et al., 2024)	10, 20 mg/kg/day, intraperitoneal injections for 2 weeks	si-TBC1D15, enalapril	mTOR, p-mTOR, PGC1α, CPT1A, PPARα, p62, ATG5, LC3B	Inducting mitophagy *via* the TBC1D15/Fis1/Rab7A signaling pathway	TAC surgery-induced male C57BL/6 mice model	Liu W. et al. (2026)
	Berberine	*Coptis chinensis* Franch	HF (Cao et al., 2026), myocardial infarction (Li et al., 2025), diverse vascular diseases (Ai et al., 2021), atrial fibrillation (Wang Y. et al., 2025), coronary microembolization (Wei et al., 2026), coronary heart disease (Han Y.-C. et al., 2022), DOX-induced cardiotoxicity (Wang Y.-Y.et al., 2023), viral myocarditis (Dai et al., 2021)	5 μM, medicated culture medium for 24 h; 50 mg/kg/day, intragastric administration for 4 weeks	3-MA, bafilomycin A1, si-PINK1	MHC, BNP, p62, LC3B, COXIV, VDAC, FUNDC1, PHB2, BNIP3	Upregulating PINK1/Parkin-mediated mitophagy	PE-induced NMCMs or AMCMs injury, TAC surgery-induced male C57BL/6 J mice model	Abudureyimu et al. (2020)
	Colchicine	*Colchicum autumnale* L	HF (Wu M. et al., 2025), coronary heart disease (Katira and Katira, 2022), pericarditis (Lutschinger et al., 2019), myocardial infarction (Younas et al., 2025), atherosclerosis (Wang H. et al., 2025), post-operative atrial fibrillation (Ying et al., 2023), DOX-induced cardiotoxicity (Song et al., 2018)	2.5, 5, 20 nM, medicated culture medium for 24 h; 0.1, 1 mg/kg/day, intragastric administration for 10 weeks	Chloroquine	p-PI3K, p-Akt, p-mTOR, p-ULK1, p-AMPK, PINK1, Parkin, Tom20, LC3B, p62, Beclin1, ATG5, p53	Inducing autophagy *via* promoting the degradation of autolysosome	DOX-induced hiPSc- CMs injury and Syrian hamsters model	Peng et al. (2024)
Glycosides	Paeoniflorin	*Paeonia lactiflora* Pall	HF (Liu M. et al., 2020), diabetic cardiomyopathy (Zhang et al., 2026), DOX-induced cardiotoxicity (Li J.-Z. et al., 2016), hypertension (Liu et al., 2019), myocardial infarction (Chen H. et al., 2018)	400 μM, medicated culture medium for 24 h; 10, 20, 40 mg/kg/day, intragastric administration for 4 weeks	FOS, Com C, si-Parkin	AMPKα2, PINK1, Parkin, ACSL4, GPX4, COXIV	Regulating mitophagy mediated by mitochondria-associated AMPK/Parkin/ACSL4 signaling pathway	ISO-induced H9C2 cells injury and male C57BL/6 J mice models	Wang Y.-W.-Q. et al. (2026)
	Plantamajoside	*Plantago depressa* Willd	HF, myocardial infarction (Du et al., 2023), cardiac hypertrophy (Shang et al., 2019)	10, 20, 40, 80, 100 μM, medicated culture medium for 6, 12, 24 or 48 h; 50 mg/kg/day, intragastric administration for 4 weeks	RAGE^−/−^	CD31, α-SMA, COL-I, COL-III LC3B, p62, Beclin1	Inhibiting AGEs activated-RAGE/autophagy/EndMT signaling pathway	AGEs-induced H9C2 cells injury, TAC surgery-induced male C57BL/6 J mice model	Zhang L. et al. (2023)
	Sophoricoside	*Sophora japonica* L	HF	10, 50 μM, medicated culture medium for 24 h; 80, 160 mg/kg/day, intragastric administration for 4 weeks	Not mentioned	p70S6K, p-p70S6K, 4E-BP1, p-4E-BP1, ANP, BNP, MYH7, COL-I, COL-III, CTGF, p38, p-p38, JNK, p-JNK, ERK, p-ERK, NFAT3, LaminB1, LC3B, Beclin1, p62	Activating AMPK/mTORC1-mediated autophagy	PE-induced NRCMs injury, TAC surgery-induced male C57BL/6 J mice model	Gao et al. (2020)
Polyphenols	Resveratrol	*Reynoutria japonica* Houtt	HF (Liu K. et al., 2024), hypertensive heart disease (Jojima et al., 2023), myocardial infarction (Li T.-L. et al., 2024), atherosclerosis (Hu D. et al., 2025), atrial fibrillation (Cao et al., 2024), dystrophic cardiomyopathy (Kuno et al., 2015), diabetic cardiomyopathy (Yang L. et al., 2025), DOX-induced cardiotoxicity (Chen L. et al., 2024)	8 mg/kg/day, intraperitoneal injections for 4 weeks	Not mentioned	Beclin1, LAMP1	Inhibiting autophagy through the inactivation of AMPK and restoration of ATP	TAC surgery-induced male SD rats model	Wang et al. (2015)
	Tetrahydrocurcumin	*Curcuma longa* L	HF, myocardial infarction (Zhang B. et al., 2023), septic cardiomyopathy (Zhu et al., 2022), cardiac hypertrophy (Zhang B. et al., 2020)	0.5, 1, 2 μM, medicated culture medium for 24 h; 50 mg/kg/day, intragastric administration for 4 weeks	LY294002, rapamycin	HIF-1α, Bax, Bcl-2, cleaved Caspase-3, LC3B, p62, Beclin1	Induction autophagy *via* the activation of PI3K/Akt/mTOR signaling pathway	H/R-induced H9C2 cells injury, LAC-induced male SD rats model	Chen et al. (2021)
Flavonoids	Quercetin	*Styphnolobium japonicum* (L.) Schott	HF (Long et al., 2025), septic cardiomyopathy (Zhang et al., 2025), myocardial infarction (Wang L. et al., 2025), postmenopausal atherosclerosis (Lv et al., 2026), diabetic cardiomyopathy (Khajehlandi and Bolboli, 2025), coronary heart disease (Mury et al., 2025), cardiac arrhythmia (Zhou et al., 2022), hypertension (Wu et al., 2024)	100 μM, medicated culture medium for 24 h; 100 mg/kg/day, intraperitoneal injections for 4 weeks	shp53, LV-p53	p62, LC3B	Activating autophagy through activating MDM2-dependent p53 ubiquitination and degradation	β_1_-AA-induced H9C2 cells and male C57BL/6 J mice models	Ma M. et al. (2025)
Triterpenes	Gypenosides	*Gynostemma pentaphyllum* (Thunb.) Makino	HF, diabetic cardiomyopathy (Zhang et al., 2018), pulmonary arterial hypertension (Du et al., 2025)	10, 20, 40 μg/mL, medicated culture medium for 24 h	Not mentioned	MFN1, MFN2, Drp1, Fis1, PINK1, Parkin, LC3B	Promoting mitophagy through the activation of PI3K/Akt/GSK-3 β/Mcl-1 signaling pathway	DOX-induced H9C2 cells injury model	Zheng et al. (2024)
Saponins	Astragaloside IV	*Astragalus membranaceus* (Fisch.) Bunge	HF (Wang et al., 2024), septic cardiomyopathy (Wang J. et al., 2025), myocardial infarction (Shi et al., 2021), radiation-induced heart disease ( Li Q. et al., 2025), hypertensive heart disease (Jing et al., 2025), diabetic cardiomyopathy (Dong et al., 2026), DOX-induced cardiotoxicity (Tian et al., 2024), viral myocarditis (Wang M. et al., 2025)	50 μM, medicated culture medium for 24 h; 20, 80 mg/kg/day, intragastric administration for 8 weeks	SB203058	PGC-1α, TFAM, Parkin, p62, Beclin1, LC3B, p38, p-p38	Promoting mitophagy through the inhibition of MAPK signaling pathway	DOX-induced H9C2 cells injury, AAC surgery-induce male SD rats model	Wang Y. et al. 2026
Aldehydes	Cinnamaldehyde	*Cinnamomum cassia* (L.) D. Don	HF (Xu et al., 2024), myocardial infarction (Yang M. et al., 2025), diabetic cardiomyopathy (Hu M.-Q. et al., 2025), ventricular arrhythmia (Ma G. et al., 2025), DOX-induced cardiotoxicity (Mao et al., 2023), atherosclerosis (Li W. et al., 2019), viral myocarditis (Ding et al., 2010)	50 mg/kg/day, intragastric administration for 4 weeks	Captopril	p62, LC3B	Activating autophagy through the inhibition of mTOR signaling pathway	STZ injection-induced male diabetic rats model	Hu M.-Q. et al. (2025)

TCM, traditional chinese medicine; HF, heart failure; DOX, doxorubicin; TCA, transverse aortic constriction, 3-MA, 3-Methyladenine; PE, phenylephrine; FOS, fosinopril; ISO, isoproterenol, H/R, hypoxia and reoxygenation, LAC, left coronary artery, β_1_-AA, β_1_-adrenergic receptor autoantibody, SD, Sprague-Dawley; AAC, abdominal aortic constriction; STZ, streptozotocin.

**TABLE 4:** Active metabolites of Chinese herbal compound preparations and single-flavored TCM extracts *via* mass spectrometry or network pharmacology technology.

Chinese herbal compound preparation/Single-flavored TCM extract	Mass spectrometry/Network pharmacology	Active metabolite	References
Huangqi Danshen decoction	UPLC-MS/MS	Danshensu, calycosin-7-O-β-d-glucoside, salvianolic acid C, ononin, lithospermic acid, salvianolic acid B, salvianolic acid A, isomucronulatol 7-O-glucoside, calycosin, formononetin, tashinone IIA	Chen Z. et al. (2024)
Ershen Zhenwu decoction	UHPLC-Q-TOF-MS	Saponins, terpenoids, alkaloids, phenolic acids, tanshinones, ureas, aromatic hydrocarbons, alkanes, steroidal metabolites	Wang X. et al. (2026)
Linggui Zhugan decoction	Network pharmacology (TCM Systems Pharmacology and Analysis Database	Quercetin, naringenin, 1-methoxyphaseollidin	Ren et al. (2024)
Si-Miao-Yong-An decoction	UPLC-Q/TOF-MS	Angoriside C, 3,5-dicaffeoylquinic acid	Liao et al. (2022)
Qili Qiangxin capsule	UPLC-MS	Astragaloside, calycosin-7-0-glucoside, ginsenoside Rb1, ginsenoside Re, ginsenoside Rd, ginsenoside Rg1, ginsenoside Rf, periplocin, periplocoside H1, hesperidin, narirutin, isoquercitrin	Liu et al. (2014)
Shengxian Sanren granules	UPLC-Q-TOF-MS/MS	Sucrose, quinic acid, catechol, ferulic acid, azelaic acid, formononetin, ginsenoside Rb1, astragaloside IV, 16-oxo-alisol A, alisol A, poricoic acid B	Dai et al. (2025)
Shenqi oral liquid	UPLC-Q-TOF-MS/MS	Flavonoids, saponins, terpenoids, acids, acetylene metabolites, amino acid metabolites, alkaloid metabolites, flavan metabolites, phenylpropanoid metabolites	Xie et al. (2026)
*Panax ginseng* C. A. Mey. extract	UHPLC-PDA	Ginsenoside Re, ginseno side Rg1, ginsenoside Rf1, ginsenoside Rb1	Dou et al. (2024)
*Crocus sativus* L. extract	HPLC-UV, HPLC-MS, TLC	Hydroxysafflor yellow A, kaempferide, nicotiflorin	Chen J. et al. (2024)
*Astragalus membranaceus* (Fisch.) Bunge injection	HPLC-ESI-TOF-MS/MS	Calycosin 7-O-β-D-glucoside, ononin, methylnissolin-3-O-glucoside, astraisoflavan-7-O-β-D-glucoside, calycosin, astragaloside IV, formononetin, astragaloside II, isoastragaloside II, astragaloside I, isoastragaloside I	Liu H.-X. et al. (2025)
Total glucosides of *Paeonia lactiflora* Pall	HPLC-MS/MS	Gallic acid, hydroxypaeoniflorin, catechin, albiflorin, paeoniflorin, pentagalloyl glucose, benzoic acid, benzoylpaeoniflorin, paeonol	Jiang et al. (2021)
Total saponin of *Scrophularia ningpoensis* Hemsl	UPLC-Q-TOF/MS	Ginsenoside Rg1, ginsenoside Re, ginsenoside Rb1, ginsenoside Rh1, ginsenoside Rg3, ginsenoside Rh2	Li L. et al. (2023)
*Aconitum carmichaelii* Debeaux extract	UHPLC-Q-TOF-MS	Linoleic, palmitic, oleic acid, fatty acid	Liang et al. (2018)

TCM, traditional chinese medicine.

**TABLE 5:** Structural information and pharmacological effects of active metabolites in TCM.

Active metabolite	2D structure	Molecular formula	Molecular weight	PubChem CID	Chemical category	Pharmacological effects	References
Songorine	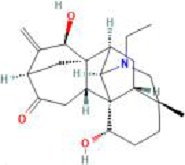	C_22_H_31_NO_3_	357.50 g/mol	71456946	Alkaloids	Cardiovascular protection, anti-cancer, anti-depressant, anti-nociceptive, anti-inflammatory, anti-pyretic, anti-platelet, antioxidant, antibacterial	Khan et al. (2018)
Berberine	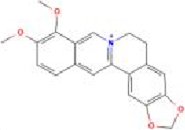	C_20_H_18_NO_4_^+^	336.40 g/mol	2353	Alkaloids	Cardiovascular protection, neuroprotection, metabolic regulation, anti-inflammation, anti-cancer, antioxidant, anti-apoptotic, anti-ischemic	Song et al. (2020)
Colchicine	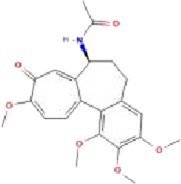	C_22_H_25_NO_6_	399.40 g/mol	6167	Alkaloids	Cardiovascular protection, skin care, anti-gout, anti-inflammatory, anti-diabetes, immune-regulation, anti-stroke	Lunzer et al. (2025)
Paeoniflorin	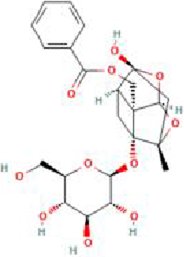	C_23_H_28_O_11_	480.50 g/mol	442534	Glycosides	Cardiovascular protection, anti-inflammation, antioxidant, anti-thrombotic, anti-convulsive, analgesic, neuroprotection, liver protection, anti-depressant, anti-cancer, immune-regulation	Zhou et al. (2020)
Plantamajoside	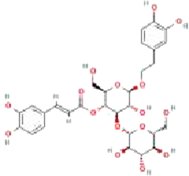	C_29_H_36_O_16_	640.60 g/mol	5281788	Glycosides	Cardiovascular protection, neuroprotection, anti-cancer, anti-inflammatory, antioxidant	Shang et al. (2019), Hai et al. (2025), Zuo et al. (2021), Jia et al. (2025), Zeng et al. (2022)
Sophoricoside	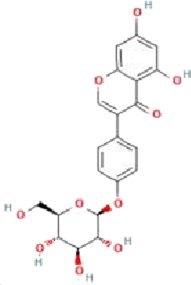	C_21_H_20_O_10_	432.40 g/mol	5321398	Glycosides	Cardiovascular protection, anti-inflammatory, liver protection, neuroprotection, anti-cancer	Xu and Hao (2026), Kim and Lee (2021), Zheng et al. (2026), Wang H.-F. et al. (2023), Wang J.-Z. et al. (2026)
Resveratrol	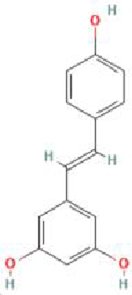	C_14_H_12_O_3_	228.24 g/mol	445154	Polyphenols	Cardiovascular protection, antibacterial, anticancer, neuroprotective	Tian and Liu (2020)
Tetrahydrocurcumin	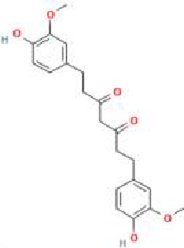	C_21_H_24_O_6_	372.40 g/mol	124072	Polyphenols	Cardiovascular protection, anti-inflammatory, metabolic regulation, neuroprotection, anti-cancer	Zhou M. et al. (2024)
Quercetin	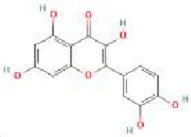	C_15_H_10_O_7_	302.23 g/mol	5280343	Flavonoids	Cardiovascular protection, neuroprotection, antibacterial, anti-inflammatory, anticancer, antioxidant	Alizadeh and Ebrahimzadeh (2022)
Gypenosides	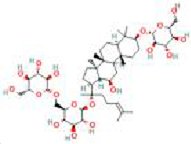	C_48_H_82_O_18_	602.80 g/mol	44584555	Triterpenes	Cardiovascular protection, anti-cancer, neuroprotective, anti-anxiety, anti-depressant, anti-diabetic	Su et al. (2021)
Astragaloside IV	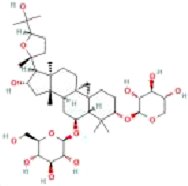	C_41_H_68_O_14_	785.00 g/mol	13943297	Saponins	Cardiovascular protection, anti-inflammatory, anti-fibrotic, antioxidant, anti-cancer, neuroprotective	Liang et al. (2023)
Cinnamaldehyde	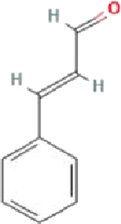	C_9_H_8_O	132.16 g/mol	637511	Aldehydes	Cardiovascular protection, anti-inflammatory, anti-tumor, antibacterial, anti-diabetes, anti-obesity, neuroprotective	Zhang et al. (2015)

TCM, traditional chinese medicine.

**TABLE 6:** Summary of clinical studies on representative botanical drugs in cardiovascular diseases.

Botanical drugs	Cardiovascular diseases	Study design and participants	Treatment regimen	Main outcomes	Main findings	Adverse events	References
HDD	Coronary heart disease	RCT involving 160 patient	Combining with standard therapy for 28 days	hs-CRP, MCP-1, IL-6, IL-1β, IL-8, IL-10, TNF-α, OX-LDL, NT-proBNP, cTn, CK-MB, H-FABP,	Improving damage from myocardial ischemia and reducing inflammation	No serious adverse events reported	(Luo et al., 2020)
Fuyu Decoction	CHF	RCT involving 112 patients	Combining with standard therapy for 6 months	LVEF, LVESD, LVEDD, TGF-β1, CTGF, NT-proBNP, IL-6, TNF-α, Ang-II, ET-1, NO	Improving heart function, protecting the blood vessel lining, slowing down myocardial fibrosis	No serious adverse events reported	Wang Q.-Q. et al., 2025
ESZWD	HFrEF and HFmrEF	RCT involving 114 patients	Combining with standard therapy for 8 weeks	LVEF, LVIDd, LVIDs, IVSd, LVPWd, LVFS, NT-proBNP, Ang-II,	Alleviating myocardial fibrosis by regulating the miR-423-5p/Smad7/TGF-β1/Smads axis and suppressing α-SMA and Fn expression	No serious adverse events reported	(Ge et al., 2025)
GS	ST-elevation acute myocardial infarction	RCT involving 50 patients	Combining with CFR therapy for 8 months	CD34+ , CXCR4+, CD117+, CD133+, C-met+, IL-6, TNF-α, hs-CRP	Increaseing the circulating angiogenic cell mobilization and decreasing inflammation	No serious adverse events reported	(Ahn et al., 2011)
Crocus sativus L. extract	Unstable angina pectoris	Meta-analysis of 7 RCTs involving 1134 patients	Combining with standard therapy for 3 months	Relief rate of angina symptoms, ECG	Showing advantages in easing the clinical symptoms of unstable angina and improving ECG over basic therapy alone	No serious adverse events reported	(Kong et al., 2013)
AMI	HFrEF	Meta-analysis of 19 RCTs involving 1565 patients	Combining with standard therapy therapy for 8 weeks	LVEF, LVEDD, LVESD, NT-proBNP, 6-MWD, TNF-α, IL-6, hs-CRP	Having more advantages than CT alone in improving LVR and clinical efficacy in HFrEF patients	No serious adverse events reported	(Han et al., 2024)
SGR	CHF	Population-based PS-matched cohort study involving 1842 patient	Combining with standard therapy therapy for 3 months	All-cause mortality, composite cardiovascular	Having a significant benefit in preventing cardiovascular events	No serious adverse events reported	(Tai et al., 2022)
PF	Restenosis following percutaneous coronary intervention	RCT involving 335 patient	Combining with standard therapy for 6 months	Restenosis rates, MLD, combined incidence of major adverse cardiac event	Showing the safe and effective in reducing restenosis in post-PCI patients	No serious adverse events reported	(Chen et al., 2006)
Resveratrol	Hypertension	Meta-analysis of 17 RCTs	Combining with standard therapy for 6 months	SBP, DBP, BMI	Promoting cardiovascular health, mostly when used in high daily dose (≥300 mg/day) and in diabetic patients	No serious adverse events reported	(Fogacci et al., 2019)

HDD, huangqi danshen decoction; RCT, randomized controlled trial; CHF, chronic heart failure; ESZWD, ershen zhenwu decoction, HFrEF, heart failure with reduced ejection fraction, HFmrEF, Heart failure with mid-range ejection fraction; GS, ginseng radix et rhizoma; CFR, coronary flow reserve; AMI, astragalus membranaceus injection; SGR, songorine; PS, propensity score; PF, paeoniflorin.

### 6.1 Chinese herbal compound preparations delay HF progression by targeting autophagic flux

Through the rational compatibility of multiple botanical drugs, Chinese herbal compound preparations have distinctive advantages such as synergistic efficacy, reduced toxicity, and enhanced therapeutic effects. These properties make them particularly valuable in the treatment of complex diseases such as HF. The pathogenesis of HF involves multiple factors and mechanisms, including disordered cardiomyocyte energy metabolism, mitochondrial dysfunction, pathological myocardial hypertrophy and fibrosis, oxidative stress, and sterile inflammation. Therefore, Chinese herbal formulas with multi-metabolite, multi-target, and multi-pathway regulatory characteristics are increasingly regarded as important strategies for clinical intervention in HF. Representative compound preparations that treat HF by regulating key autophagy pathways have been identified, including Huangqi Danshen Decoction (HDD), Fuyu Decoction, Ershen Zhenwu Decoction (ESZWD), Linggui Zhugan Decoction, Qili Qiangxin capsule, Si-Miao-Yong-An Decoction, Shengxian Sanren granules, and Shenqi oral liquid. Among them, HDD, Fuyu Decoction, and ESZWD best reflect the synergistic mechanisms of multidimensional intervention in HF.

*Astragalus membranaceus* (Fisch.) Bge. *and Salvia miltiorrhiza* Bge. *c*onstitute a representative botanical drug pair for replenishing qi and activating blood, with anti-myocardial remodeling and anti-inflammatory effects (Wang X.-D. et al., 2017). Previous studies have shown that HDD can activate AMPK and improve myocardial remodeling, providing a new perspective for AMPK-targeted therapy in HF (Wang et al., 2017). In recent years, the role of pericardial adipose tissue (PAT) in cardiovascular disease has attracted increasing attention, but whether the specific mechanism by which HDD improves HF is related to PAT remains unclear. To address this question, Chen Z-Y *et al.* (Chen Z. et al., 2024) investigated the role of PAT-derived extracellular vesicles (EVs) in HF and elucidated the protective effect of HDD on myocardial remodeling, along with its potential molecular mechanism. Their findings demonstrated that HDD improved cardiac function and alleviated myocardial hypertrophy and fibrosis in HF rats. Mechanistically, HDD suppressed the secretion of miR-27a-3p in PAT-derived EVs. This miRNA was found to negatively regulate AMPKα2 expression by targeting PRKAA2, thereby inhibiting PINK1/Parkin-mediated mitophagy. By reducing miR-27a-3p levels in PAT-EVs, HDD activated the AMPK/PINK1/Parkin signaling axis, which enhanced mitophagy, improved cardiomyocyte energy metabolism, attenuated myocardial remodeling, and ultimately alleviated HF.

In recent years, TCM has made notable progress in the treatment of CHF and has gained broad recognition for its definite efficacy and favorable safety profile (Xu et al., 2022). *Astragalus membranaceus* (Fisch.) Bge. *and Salvia miltiorrhiza* Bge., composed of *Cornus officinalis* Siebold & Zucc., *Aconitum carmichaelii* Debx., and *Glycyrrhiza uralensis* Fisch., is formulated to replenish qi, warm yang, resolve blood stasis, and unblock collaterals. Previous studies have shown that Fuyu Decoction combined with conventional Western medicine significantly improves clinical symptoms and ventricular function, delays ventricular remodeling, and achieves satisfactory therapeutic effects in HF patients (Wang et al., 2025), suggesting its potential as a promising herbal compound for HF. However, the underlying mechanism remains unclear. Given that AMPK/mTOR-mediated autophagy plays a critical role in ventricular remodeling during HF (Fan et al., 2022; Zhang X. et al., 2019). Against this background, Ma J *et al.* (Ma et al., 2023) investigated whether Fuyu Decoction ameliorates ventricular remodeling through regulation of this autophagic pathway. Their findings demonstrated that Fuyu Decoction effectively improved cardiac function, reduced myocardial infarct size, attenuated myocardial fibrosis and pathological lesions, and decreased cardiomyocyte apoptosis in HF rats. Mechanistically, Fuyu Decoction suppressed the AMPK/mTOR signaling pathway, as evidenced by decreased p-AMPK levels and increased p-mTOR expression, while concurrently modulating autophagy-related markers (reduced LC3-II/LC3-I ratio and Beclin1, increased p62), indicating inhibition of excessive autophagy. Notably, these beneficial effects were reversed by co-administration of an AMPK agonist (EX229), confirming that the protective action of Fuyu Decoction is mediated through inhibition of AMPK/mTOR-driven autophagy. Collectively, these results indicate that Fuyu Decoction alleviates ventricular remodeling in HF by inhibiting AMPK/mTOR signaling-mediated autophagy in cardiomyocytes.

ESZWD is derived from Zhenwu Decoction, a classical formula recorded in the Treatise on Cold Damage, with the addition of *Panax ginseng* C. A. Mey. to replenish qi and consolidate blood, and *Salvia miltiorrhiza* Bge. to activate blood and resolve stasis. According to TCM theory, *A. carmichaelii* Debx. and *P. ginseng* C. A. Mey. serve as sovereign agents to warm yang, replenish qi, tonify heart-kidney yang, and promote blood circulation. *Poria cocos* (Schw.) Wolf and *Atractylodes macrocephala* Koidz. act as ministerial herbs to strengthen the spleen, drain dampness, and promote diuresis. *Cynanchum otophyllum* Schneid. moderates the drying properties of the sovereign herbs to prevent yin injury. *Salvia miltiorrhiza* Bge. also activates blood and resolves stasis to unblock vessels. *Zingiber officinale* Roscoe serves as an adjuvant, supporting the sovereign herbs in warming and tonifying spleen-kidney yang and harmonizing the formula. Collectively, ESZWD replenishes qi, warms yang, activates blood, and promotes diuresis, combining supplementation with dispersion and warming with transformation. Several of its metabolites have dual medicinal and food properties, and the formula has been used clinically for over 2 decades. Previous clinical studies have shown that ESZWD significantly improves cardiac function, relieves symptoms, and enhances quality of life in CHF patients (Ge et al., 2025). *In vivo* and *in vitro* experiments have confirmed its cardioprotective effects through reducing oxidative stress, inhibiting cardiomyocyte apoptosis, and attenuating myocardial fibrosis (Cheng et al., 2025; Liu Y. et al., 2025). However, whether ESZWD improves CHF *via* inhibition of mitophagy remained unclear. To address this gap, Wang X-Y *et al.* (Wang X. et al., 2026) investigated the role of the PINK1/Parkin signaling pathway in mediating the effects of ESZWD. Their findings demonstrated that ESZWD treatment effectively improved cardiac function, reduced myocardial injury, and alleviated pathological changes in CHF models. Mechanistically, ESZWD regulated the expression of key mitophagy-related proteins (PINK1, Parkin, LC3-II/I, Beclin-1, and p62), indicating suppression of pathological mitophagy. Further validation confirmed that the inhibitory effect of ESZWD on mitophagy was comparable to that of an autophagy inhibitor 3-methyladenine (3-MA), supporting its action through the PINK1/Parkin axis. Collectively, these results suggest that ESZWD alleviates CHF by inhibiting PINK1/Parkin-mediated mitophagy.

### 6.2 Single-botanical drug extracts delay HF progression by targeting autophagic flux

Natural herbal extracts have attracted considerable attention in HF treatment because of their multi-target regulatory effects and high biocompatibility. For example, extracts from medicinal plants such as *P. ginseng* C. A. Mey., *Crocus sativus* L., *A. membranaceus* (Fisch.) Bunge., *Paeonia lactiflora* Pall., *Scrophularia ningpoensis* Hemsl. and *A. carmichaelii* Debeaux all play important roles in regulating cardiomyocyte autophagy. Among them, active extracts from *P. ginseng* C. A. Mey., *C. sativus* L., *A. membranaceus* (Fisch.) Bunge. have shown the most notable research progress in HF treatment.

*Panax ginseng* C. A. Mey., known as the “king of botanical drugs,” is mainly cultivated in China, Korea, and the United States. Historically, its medicinal use was attributed to the root, Ginseng Radix et Rhizoma (GS). Today, its applications have expanded to leaves, stems, fruits, and flowers (Ito and Ito, 2024). GS has been used for approximately 2,000 years, with the earliest record in Shennong’s Classic of Materia Medica. In China, GS is included in the Catalogue for the Management of Traditional Foods and Chinese Medicinal Materials, and the United States Pharmacopeial Convention recognizes it as a dietary supplement. GS contains diverse active metabolites, notably ginsenosides, polysaccharides, polyacetylenes, amino acids, and phenolic metabolites (Liu L. et al., 2024). Among these, ginsenosides are the most abundant and extensively studied (Liu H. et al., 2020), and serve as quality control markers in both the Chinese and Japanese pharmacopoeias. Modern research has demonstrated that GS and its preparations exhibit multiple pharmacological activities, including anticancer, immunomodulatory, anti-inflammatory, anti-allergic, anti-atherosclerotic, antihypertensive, antidiabetic, anti-stress, antioxidant, and anti-apoptotic effects (Liu H. et al., 2022; Yang Y. et al., 2024). Owing to these broad benefits, GS formulations (oral liquids, tablets, and injections) have been officially registered and marketed, showing clinical efficacy in cardiovascular diseases, immune modulation, glucose-lipid metabolism, fatigue relief, cancer support, inflammation, pain, and aging delay. To date, no reports have addressed the combined use of GS and metoprolol in HF. Therefore, Niu Z-C *et al.* (Zi-Chang et al., 2024) investigated this combination and its underlying mechanism. Their findings demonstrated that GS combined with metoprolol improved cardiac function, attenuated myocardial injury, and reduced serum biomarkers of cardiac damage in HF models. Mechanistically, the combination inhibited cardiomyocyte autophagy, as evidenced by decreased expression of autophagy-related proteins (LC3, p62, Beclin1, ATG5) and increased phosphorylation ratios of PI3K, Akt, and mTOR, indicating activation of the PI3K/Akt/mTOR signaling pathway. These results suggest that GS enhances the anti-HF effect of metoprolol through suppression of autophagy *via* PI3K/Akt/mTOR activation.

*Crocus sativus* L., known as “red gold,” is mainly cultivated in Iran, India, Spain, and Greece, and has a medicinal history of approximately 3,000 years, with the earliest records found in ancient Persian and Greek medical texts. It contains multiple active metabolites, including saffronin, saffronic acid, bitter saffronin, volatile oils, and phenolic metabolites (Xing et al., 2021). Among these, saffronin is the most abundant and extensively studied (Hosseini et al., 2018), and serves as a quality control marker in the Chinese Pharmacopoeia. Recent studies have demonstrated that *C. sativus* L. and its preparations exhibit multiple pharmacological activities, including neuroprotective, antioxidant, antidepressant, anxiolytic, antitumor, anti-inflammatory, and anti-apoptotic effects (Marrone et al., 2024). Owing to these broad benefits, this botanical drug is used to treat various diseases and plays an important role in health promotion. To date, no reports have described the use of saffron in patients with HF. Therefore, Jia Z-S *et al.* (Jia et al., 2015) investigated the effects of *C. sativus* L. extract on cardiomyocyte injury. Their findings demonstrated that the extract inhibited apoptosis in injured cardiomyocytes. Mechanistically, the extract induced autophagy in these cells, as evidenced by increased autophagosome formation and LC3-II conversion. Importantly, the anti-apoptotic effect of the extract was abolished when autophagy was blocked, whereas stimulation of autophagy independently suppressed apoptosis. In parallel, the extract downregulated the expression of pro-apoptotic mediators (BAD and Bax). Collectively, these results indicate that *C. sativus* L. extract exerts its protective effect by inducing cardiomyocyte autophagy, thereby suppressing apoptosis, and suggest its potential as a novel therapeutic agent for HF.

*Astragalus membranaceus* is the dried root of the leguminous plant *A. membranaceus* (Fisch.) Bge. and is widely used in China as a traditional Chinese medicinal botanical drug for treating cardiovascular diseases (Kanaan et al., 2017; Liu K. et al., 2022). Astragalus membranaceus injection (AMI), a patented Chinese medicine extract prepared by water-extraction and alcohol-precipitation, has been approved by the China Food and Drug Administration (CFDA). Numerous studies have demonstrated the efficacy of Astragalus membranaceus in patients with CHF (Han et al., 2024). A systematic review and meta-analysis of 16 randomized controlled trials showed that AMI combined with Western medicine improved left ventricular ejection fraction (LVEF) in CHF patients (Wang K. et al., 2017). Although the mechanisms of action of its active metabolites have been extensively studied, the pharmacological effects of AMI in CHF remain incompletely understood. To address this gap, Li S-N *et al.* (Li et al., 2025) investigated the effects of AMI on mitophagy and mitochondrial function in CHF. Their findings revealed that AMI dose-dependently inhibited pathological cardiomyocyte hypertrophy, reduced apoptosis, restored mitochondrial function, and enhanced mitophagic flux. *In vivo*, AMI treatment significantly improved cardiac function, attenuated cardiac hypertrophy, and reduced interstitial fibrosis, while also increasing myocardial mitochondrial numbers and enhancing autophagic activity. Mechanistically, phosphoproteomic and network pharmacology analyses identified the PI3K/Akt/mTOR signaling axis as the principal regulatory pathway mediating the cardioprotective effects of AMI. Functional validation confirmed that AMI acted similarly to an Akt/mTOR inhibitor GSK-690693, effectively restoring mitophagy and mitochondrial homeostasis, whereas activation of this pathway reversed these benefits. Collectively, these results indicate that AMI exerts cardioprotective effects by inhibiting Akt/mTOR signaling, thereby ameliorating maladaptive remodeling and mitochondrial dysfunction in CHF.

### 6.3 Active metabolites derived from medicinal plants delay HF progression by targeting autophagic flux

Bioactive metabolites in medicinal plants, including songorine (SGR), paeoniflorin (PF), resveratrol, berberine, colchicine, plantamajoside, sophoricoside, tetrahydrocurcumin, quercetin, gypenosides, astragaloside IV, and cinnamaldehyde, play key roles in delaying HF progression. These metabolites regulate autophagy through multiple mechanisms, such as enhancing the clearance of damaged mitochondria by cardiomyocytes and suppressing cell death mediated by excessive autophagy, thereby providing an important theoretical basis for the development of novel anti-HF therapies. Alkaloids, glycosides, polyphenolic, and flavonoids metabolites are widely found across diverse plant families and are by no means unique to TCM. Moreover, as shown in Table 3, these compounds have been extensively investigated. On the basis of these two considerations, the present review concentrates on the pharmacological actions of these metabolites in HF, irrespective of their botanical or cultural origins, and takes SGR, PF, and resveratrol as representative examples to explore the mechanisms through which they modulate autophagy-related signaling pathways to counteract HF pathogenesis.

SGR is an alkaloid metabolite extracted from *A. carmichaelii* Debeaux, and recent studies have confirmed its pharmacological activity and safety (Guo et al., 2020). SGR exhibits multiple pharmacological effects, including anticonvulsant, anxiolytic, antiarrhythmic, stimulant, and anti-inflammatory activities (Khan et al., 2018), and has therefore attracted increasing attention. To date, nearly 450 alkaloids have been isolated from *Aconitum* plants (He et al., 2024), among which SGR demonstrates high bioavailability and wide tissue distribution (Dong et al., 2015). Owing to these properties, SGR has drawn broad interest as a potential candidate for treating cardiac diseases. For instance, SGR has been shown to increase survival in septic cardiomyopathy by promoting mitochondrial biogenesis through the PGC1α/Nrf2 signaling pathway and reducing oxidative stress (Li et al., 2021), and it can also regulate nicotinamide adenine dinucleotide (NAD)-related genes to maintain the ROS homeostasis (Bergler-Klein et al., 2014). However, the specific therapeutic mechanism of SGR in CHF remains unclear. To address this gap, Liu W-X *et al.* (Liu et al., 2026) investigated the effects of SGR in CHF. Their findings demonstrated that SGR treatment significantly improved cardiac function, reduced autophagic activity, and restored mitochondrial integrity in CHF models. Mechanistically, SGR modulated energy metabolism by altering the spatial distribution of fatty acylcarnitines, metabolites linked to β-oxidation. Furthermore, SGR enhanced mTOR phosphorylation and may have attenuated the abnormal mitochondrial-lysosomal separation *via* the TBC1D15/Fis1/Rab7A signaling pathway. These results indicate that SGR alleviates pressure overload-induced CHF through regulation of energy metabolism, attenuation of mitochondrial injury, and restoration of mitochondrial-lysosomal crosstalk. Collectively, this study provides important insights into SGR-mediated management of CHF *via* TBC1D15-related mitochondrial dysfunction.

PF is a monoterpene glycoside derived from the root of *P. lactiflora* Pall., known for its anti-inflammatory, antioxidant, and cardioprotective effects (Li T. et al., 2024). Multiple studies have shown that PF activates AMPK in various cell types, including cardiomyocytes, hepatocytes, neurons, and endothelial cells, thereby exerting diverse protective effects. For instance, PF reduces oxidative stress and apoptosis in cardiomyocytes *via* AMPK/Nrf2 activation (Zhang Q.- R. et al., 2024). In hepatocytes, PF improves lipid metabolism and reduces steatosis (Li et al., 2018). In neurons, PF exerts neuroprotective effects in cerebral ischemia models by activating AMPK (Zhang et al., 2017). And in endothelial cells, PF upregulates antioxidant enzymes and restores redox balance (Chen J. et al., 2018). Despite extensive research on AMPK in energy metabolism, mitophagy, and lipid metabolism, the integrated contribution of these processes to cardioprotection remains unclear. In particular, whether PF exerts cardioprotective effects by regulating mitophagy and lipid metabolism—and how this regulation affects mitochondrial ACSL4 expression—has not been fully elucidated. Therefore, Wang Y-W-Q *et al.* (Wang et al., 2026) investigated the therapeutic effects of PF on cardiac hypertrophy and its potential mechanisms, focusing on the AMPK/Parkin/ACSL4-mediated mitophagy and ferroptosis pathways. Their findings demonstrated that PF significantly attenuated cardiac hypertrophy and improved cardiac function *in vivo*. Mechanistically, PF activated AMPK and promoted Parkin-dependent mitophagy, while simultaneously reducing mitochondrial ACSL4 accumulation, thereby limiting ferroptosis. Targeted lipidomic analysis identified several PF-responsive metabolites associated with lipid peroxidation, and key pathway proteins involved in mitophagy and ferroptosis were found to be coordinately regulated. Importantly, the protective effects of PF were abolished when AMPK was inhibited or Parkin was knocked down, confirming the specificity of the AMPK/Parkin/ACSL4 signaling axis. Additionally, PF reduced mitochondrial ROS production and iron overload, further supporting its regulatory role in ferroptotic signaling. Collectively, these results indicate that PF alleviates cardiac hypertrophy by stimulating AMPK-dependent mitophagy and inhibiting ferroptosis through the Parkin/ACSL4 signaling pathway. This study clarifies the molecular basis of PF-mediated cardioprotection and supports its potential application in treating abnormal cardiac remodeling.

Resveratrol is a polyphenolic metabolite with established cardioprotective effects in cardiovascular diseases such as cardiac hypertrophy and myocardial ischemia/reperfusion (Burstein et al., 2007; Opie and Lecour, 2007; Robich et al., 2010). Studies have shown that resveratrol can prevent HF development and improve cardiac dysfunction (Rimbaud et al., 2011; Wojciechowski et al., 2010). However, its regulatory effects on autophagy vary across different cardiovascular disease models. In mice with extensive old myocardial infarction, resveratrol reverses cardiac remodeling by enhancing autophagy (Kanamori et al., 2013). Similarly, in mice with diabetic cardiomyopathy, it enhances autophagy and improves cardiac dysfunction (Wang et al., 2014). In contrast, in mice with HF secondary to myocardial ischemia, resveratrol suppresses autophagy and attenuates cardiac remodeling (Xuan et al., 2012). Currently, the effects of resveratrol on autophagy in pressure overload-induced HF remain unclear, and whether it alleviates cardiac dysfunction by suppressing maladaptive autophagy has yet to be established. Therefore, Wang L-J *et al.* (Wang et al., 2015) investigated the effects of resveratrol in a pressure overload-induced HF model. Their findings demonstrated that resveratrol treatment significantly improved cardiac dysfunction and reduced brain natriuretic peptide (BNP) expression. Mechanistically, resveratrol downregulated autophagy-related proteins (Beclin1 and LAMP-1) and inhibited autophagic vacuole formation in failing hearts. In parallel, resveratrol restored myocardial ATP levels and reduced AMPK phosphorylation at Thr172. Collectively, these results suggest that in pressure overload-induced HF, resveratrol inhibits autophagy by restoring ATP levels and suppressing AMPK activation. This study indicates that resveratrol may have potential therapeutic benefits for patients with hypertensive heart disease.

The three categories of interventions—compound prescriptions, single-botanical drug extracts, and active metabolites—each offer distinct advantages and face specific limitations in the context of HF treatment. Compound prescriptions (e.g., HDD, Fuyu Decoction, ESZWD) embody the “multi-metabolite, multi-target, multi-pathway” paradigm of TCM, enabling synergistic regulation of multiple pathological processes (autophagy, mitophagy, inflammation, fibrosis, and energy metabolism) while potentially mitigating toxicity through rational compatibility. However, their inherent chemical complexity poses substantial challenges in quality control, standardization, and precise mechanistic elucidation, and the translation of empirical formulas into evidence-based therapy requires rigorous clinical validation. Single-botanical durg extracts (e.g., GS, *C. sativus* L. extract, AMI) represent an intermediate tier: they retain a broad spectrum of bioactive metabolites and have demonstrated clinical efficacy in improving cardiac function and biomarkers, with some formulations already approved as patented medicines. Nevertheless, their compositional diversity still complicates pharmacokinetic profiling and obscures the contribution of individual constituents, and batch-to-batch variability remains a concern. In contrast, active metabolites (e.g., SGR, PF, resveratrol) offer the clearest mechanistic resolution, allowing precise interrogation of specific molecular targets (e.g., AMPK, Parkin, mTOR) and facilitating structure-activity optimization. Their purity enables robust quality assurance and facilitates drug-likeness evaluation. However, monomers may lack the cooperative or buffering effects present in complex matrices, and single-target modulation may prove insufficient to counteract the multifactorial nature of HF, potentially limiting therapeutic efficacy. Additionally, the dose-dependent toxicity and long-term safety of pure compounds require extensive evaluation. Collectively, these three approaches are not mutually exclusive but complementary: compound prescriptions provide a holistic framework for synergistic intervention, single-botanical drug extracts bridge traditional use with modern pharmacology, and active monomers offer a reductionist tool for mechanism-driven drug discovery. Future strategies should integrate systems biology, network pharmacology, and rigorous clinical trial designs to leverage the strengths of each while addressing their respective weaknesses, ultimately advancing personalized and evidence-based HF therapies.

## 7 Discussion

HF is the terminal manifestation of multiple cardiovascular diseases. Its main features include dyspnea, reduced exercise tolerance, fluid retention, such as peripheral edema and pulmonary congestion, and fatigue, and it is often accompanied by systemic manifestations such as renal dysfunction and cognitive impairment. HF has now become a major public health problem worldwide, and its prevalence continues to rise. Although substantial progress has been made in current anti-HF drugs, such as ARNIs and beta-blockers, they still cannot reverse damaged myocardial structures or completely prevent disease progression. In addition, long-term medication often leads to complications such as hypotension, electrolyte disturbances, and deterioration of renal function, and some patients show drug intolerance or poor responses. Therefore, new strategies that can delay disease progression and reduce adverse effects associated with long-term treatment are urgently needed. Recent studies indicate that autophagy dysfunction is closely associated with the pathological mechanisms of HF. The key processes such as abnormal protein aggregation, mitochondrial dysfunction, and myocardial fibrosis can all be regulated through autophagic mechanisms. Based on a systematic review of the existing literature, this article discusses the possible effects and molecular mechanisms of Chinese herbal compound preparations, single-botanical drugs, and their active metabolites in combating HF by targeting autophagy regulation.

Natural medicinal plants, as fundamental sources of therapeutic agents, have attracted great attention because they are rich in bioactive metabolites and possess diverse pharmacological effects. Although these plants have historically been important and show great potential in drug discovery, transforming traditionally used or preliminarily supported plant extracts into clinically approved HF therapies still faces many challenges. First, a key bottleneck is batch-to-batch variability in herbal extracts. Because of the inherent chemical diversity of plant-derived metabolites, their composition is highly susceptible to factors such as growing conditions, including soil and climate, harvest time, extraction method, and plant genetic characteristics. This variability can cause substantial fluctuations in chemical profiles and active metabolite content across batches, directly affecting the reproducibility of preclinical studies and posing major challenges to dose reliability and therapeutic consistency. Second, the multi-metabolite nature of botanical drugs complicates full elucidation of their mechanisms of action. In addition, poor bioavailability, limited metabolic stability, and potential botanical drug interactions further hinder clinical application. Particularly important is the ability of drugs to target and accumulate in cardiac tissue, which is a critical prerequisite for candidate agents intended to treat cardiovascular diseases such as HF. Even if active metabolites demonstrate strong cardioprotective potential *in vitro* or in peripheral models, the expected therapeutic effect is difficult to achieve if they cannot effectively reach cardiomyocytes, mitochondria, or specific subcellular structures. Many studies evaluating the pharmacological activity of plant extracts often overlook this key pharmacokinetic parameter, causing many promising candidate metabolites to encounter obstacles in translational research. Therefore, to successfully integrate natural medicinal plants into evidence-based clinical practice for cardiovascular disease, future research must address several core challenges: establishing rigorous standardization procedures to control and monitor the chemical composition and variability of extracts. Applying modern analytical technologies to comprehensively clarify their pharmacokinetic characteristics, particularly the distribution and metabolic profiles of key active metabolites in myocardial tissue. And systematically conducting pharmacodynamic studies to elucidate their mechanisms of action. These efforts will provide a more reliable scientific basis for clinical application of medicinal plants. In future research, preclinical and clinical evidence must be systematically collected to comprehensively explore the distinctive advantages of TCM in the treatment of HF.

Although Chinese herbal compound preparations and their active metabolites show considerable potential in HF treatment research, the field still faces many challenges, especially the complexity of clinical translation and the limitations of current research paradigms. First, existing strategies that regulate autophagy for HF treatment largely remain at the cellular and animal-model stages, and rigorously designed clinical studies are lacking to verify their specific efficacy in patient populations. Notably, a general publication bias exists in the current literature, positive results are preferentially reported, whereas many clinical trials that fail to demonstrate efficacy or yield negative findings often remain unpublished. This neglect of negative data impedes comprehensive evaluation of the true therapeutic effects of TCM and may lead to overestimation of its therapeutic potential. Therefore, future clinical trial registration and reporting must become more transparent, with mandatory disclosure of all results regardless of their nature. Second, although the literature frequently emphasizes that Chinese medicines are “natural,” “non-toxic,” or generally safe, such statements often lack balanced and systematic assessment of potential risks. With respect to combination therapy, the risk of botanical drug interactions in patients currently taking standard HF medications such as ARNIs, beta-blockers, or diuretics cannot be ignored. Many herbal metabolites can alter the pharmacokinetic properties of these drugs by affecting hepatic drug-metabolizing enzymes, such as cytochrome P450 family members, or drug transporters. These interactions may cause fluctuations in blood drug concentrations, leading to hypotension, electrolyte disturbances, or renal impairment and reducing therapeutic efficacy. In addition, some botanical drugs are inherently toxic. For example, certain Chinese medicines containing aristolochic acid have definite nephrotoxicity and carcinogenicity, and some mineral- or plant-derived medicines may cause liver injury when used long term or in excessive doses. Even at routine doses, idiosyncratic reactions due to individual differences may cause severe hepatotoxicity or nephrotoxicity. Existing literature does not describe or warn about these risks with sufficient systematic rigor, so conclusions that appear “safe” in preclinical studies remain highly uncertain when applied to complex patient populations. Furthermore, a substantial translational gap remains between preclinical models and clinical application. Current experimental studies usually focus only on a single target or signaling pathway and validate it through gene knockout or antagonists. This approach, however, ignores the complex network properties of biological systems. Although multi-omics technologies, such as genomics and proteomics, are helping to overcome this limitation, the potential targets identified still need to be validated in models that more closely approximate human physiology. Moreover, positive effects observed in animal models are often offset in clinical trials by serious toxic side effects, including hepatotoxicity, nephrotoxicity, gastrointestinal reactions, and potential arrhythmia risks. This indicates that demonstrating efficacy solely at the cellular or animal level is insufficient. Future work should directly compare active metabolites or compound preparations of TCM with standardized treatment regimens, such as ARNIs and SGLT2 inhibitors, and should rigorously incorporate pharmacokinetic, toxicological, and drug-interaction studies. Only through such comprehensive evaluation can their clinical value be objectively assessed and safe and effective treatment options be provided for patients with HF.

Based on a systematic review of high-quality studies in recent years, the following five categories of herbal candidates show broad prospects for mechanistic investigation and preclinical development in HF treatment. (1) SGR, derived from *A. carmichaelii* Debeaux, can induce mitophagy by activating the TBC1D15/Fis1/Rab7A signaling pathway and attenuate cardiomyocyte apoptosis and oxidative stress. In a pressure overload-induced HF mouse model, SGR significantly improved cardiac function, reduced myocardial fibrosis, and increased cell survival. (2) PF, derived from *P. lactiflora* Pall., stimulates AMPK-dependent mitophagy and inhibits ferroptosis through the Parkin/ACSL4 signaling pathway. In ISO-induced HF mouse models and H9c2 cardiomyocyte injury models, this metabolite effectively reduces lipid peroxidation, suppresses cardiomyocyte apoptosis, and delays ventricular remodeling. (3) Resveratrol, derived from P*anax ginseng* C.A. Mey, restores myocardial ATP levels and inhibits AMPK activation, thereby suppressing autophagy. In abdominal aortic constriction-induced HF rat models, resveratrol improves LVEF and reduces BNP expression. (4) Mass spectrometry and multi-omics integration strategies have revealed that key functional metabolite groups contained in compound preparations such as HDD, Fuyu Decoction, and ESZWD may synergistically regulate autophagy or mitophagy through AMPK/mTOR and PINK1/Parkin signaling pathways, thereby exerting anti-HF effects. (5) Extracts of single medicinal plants, such as P*anax ginseng* C.A. Mey, Crocus sativus L., *A. membranaceus* (Fisch.) Bunge, *C. otophyllum* Schneid., *S. ningpoensis* Hemsl., and *A. carmichaelii* Debeaux, can regulate autophagy by targeting PI3K/Akt/mTOR, MAPK/ERK, and PARP-1 signaling pathways, reducing cardiac inflammation and improving energy metabolism. To further understand the mechanisms of action of these candidate drugs and promote their clinical translation, the following key experiments are needed. First, advanced models, such as human induced pluripotent stem cell (iPSC)-derived cardiomyocytes, especially patient-derived cells carrying HF-related gene mutations such as *MYBPC3*, *TTN*, or *LMNA*, and cardiac organoids, should be used to verify candidate efficacy and clarify their autophagy-regulatory mechanisms in complex cellular microenvironments. Second, systems pharmacology or artificial intelligence methods, such as molecular dynamics simulations and fusion-algorithm-based network pharmacology, should be applied to analyze the mechanisms of action of compound formulas or monomers and identify key functional metabolite groups and core regulatory pathways. Third, clinical trials should incorporate biomarkers of autophagy and HF progression, including monitoring of the peripheral blood LC3-II/LC3-I ratio, p62 levels, and plasma BNP/NT-proBNP and sST2. These randomized controlled trials should stratify patients according to genetic background, such as hypertrophic cardiomyopathy-related gene mutations, or HF subtype, preserved or reduced ejection fraction, to accurately quantify pharmacodynamic responses to autophagy regulation. It is important to note that the *in vitro* data cited in this review may be influenced by pan-assay interference compounds (PAINS) effects and should therefore be considered suggestive rather than conclusive (Pedro et al., 2021). Additionally, we must acknowledge that a significant portion of the current evidence is derived from *in vitro* experiments, and the potential interference of PAINS considerably limits the translational value of these findings. Consequently, rigorous validation through well-designed *in vivo* studies and large-scale clinical trials is urgently needed. By systematically screening candidate drugs, validating results using advanced models, and employing biomarker-guided clinical trial designs, the scientific basis for TCM in treating HF *via* autophagy regulation can be progressively clarified. This approach will ultimately provide a theoretical foundation and a feasible translational pathway for the modernization of TCM and the development of anti-HF therapies.
